# Synthesis of Aminoglycoside-2′-*O*-Methyl Oligoribonucleotide Fusions

**DOI:** 10.3390/molecules22050760

**Published:** 2017-05-08

**Authors:** Lotta Granqvist, Andrzej Kraszewski, Ville Tähtinen, Pasi Virta

**Affiliations:** 1 Department of Chemistry, University of Turku, Vatselankatu 2, 20014 Turku, Finland; ltgran@utu.fi (L.G.); vpotah@utu.fi (V.T.); 2 Centre of New Technologies, University of Warsaw, Banacha 2c, 02 097 Warsaw, Poland; andrzej.kraszewski@student.uw.edu.pl; 3 College of Inter-Faculty Individual Studies in Mathematics and Natural Sciences, University of Warsaw, Banacha 2c, 02 097 Warsaw, Poland

**Keywords:** aminoglycosides, triple helices, oligonucleotide conjugates

## Abstract

Phosphoramidite building blocks of ribostamycin (**3** and **4**), that may be incorporated at any position of the oligonucleotide sequence, were synthesized. The building blocks, together with a previously described neomycin-modified solid support, were applied for the preparation of aminoglycoside-2′-*O*-methyl oligoribonucleotide fusions. The fusions were used to clamp a single strand DNA sequence (a purine-rich strand of c-Myc promoter 1) to form triple helical 2′-*O*-methyl RNA/DNA-hybrid constructs. The potential of the aminoglycoside moieties to stabilize the triple helical constructs were studied by UV-melting profile analysis.

## 1. Introduction

Aminoglycosides are well-known small molecular ligands for a variety of RNA targets [1] (including bulges and internal loops at ribosomal decoding site [2,3,4,5], several ribozymes [6,7] and important regions of HIV RNAs [8,9,10,11]), and they also show relatively high affinities as groove binders for DNA- and RNA-triple helices and for their hybrids [12,13,14]. Thanks to these binding properties, they may be attractive conjugate groups for oligonucleotide-based probes to provide an extra binding motif in the recognition of the target DNA or RNA [15,16,17,18,19,20]. For example, neomycin has been used to enhance affinity of oligonucleotides to an α-sarcin loop RNA sequence [21] and to HIV-1 trans activation response element (TAR) models [22] via binding to known binding sites for neomycin on these RNA targets. In a favorable case, the cooperative recognition via combined small molecular binding and hybridization may take place [22]. Triple helical recognition of DNA has also been enhanced by appropriately conjugated neomycin ligands [23]. Furthermore, aminoglycoside moieties may improve cellular uptake via lipid-mediated delivery of oligonucleotides [24]. All together, these beneficial properties of the conjugated aminoglycosides may find applications in developing of modern antigene and antisense therapies.

**Figure 1:**
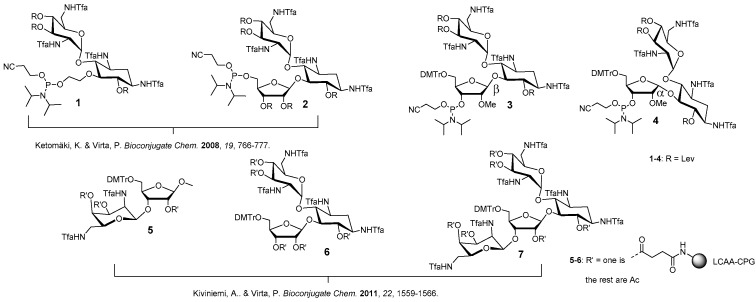
Phosphoramidite building blocks **1**–**4** and solid supports **5**–**7** useful for the automated synthesis of aminoglycoside-oligonucleotide conjugates. **3** and **4** introduced in the present study.

We have previously described aminoglycoside-derived phosphoramidite building blocks (**1** and **2**) [17] and solid supports (**5**–**7**) [22], which may be used for the automated synthesis of 5′- and 3′-aminoglycoside conjugated oligonucleotides, respectively (Figure 1). Herein, the set of the useful building blocks is expanded by appropriately modified ribostamycins (**3** and **4**) that may be incorporated at any position of the oligonucleotide sequence. The building blocks (**3** and **4**), together with a previously described neomycin-derived LCAA-CPG-support **7** (long chain alkylamine controlled pore glass), were used for the synthesis of pure fusions of the aminoglycosides and 2′-*O*-methyl oligoribonucleotides. The aminoglycoside moieties were aimed to stabilize triple helical constructs formed by clamping a purine-rich DNA single strand (a sequence of c-Myc promoter 1, [25]). The effect of the aminoglycoside moieties on the resulting triple helical 2′-*O*-methyl RNA/DNA-hybrid constructs was evaluated by UV-melting profile analysis.

## 2. Results and Discussion

### 2.1. Synthesis of the Phosphoramidite Building Blocks ***3*** and ***4***

**Scheme 1:**
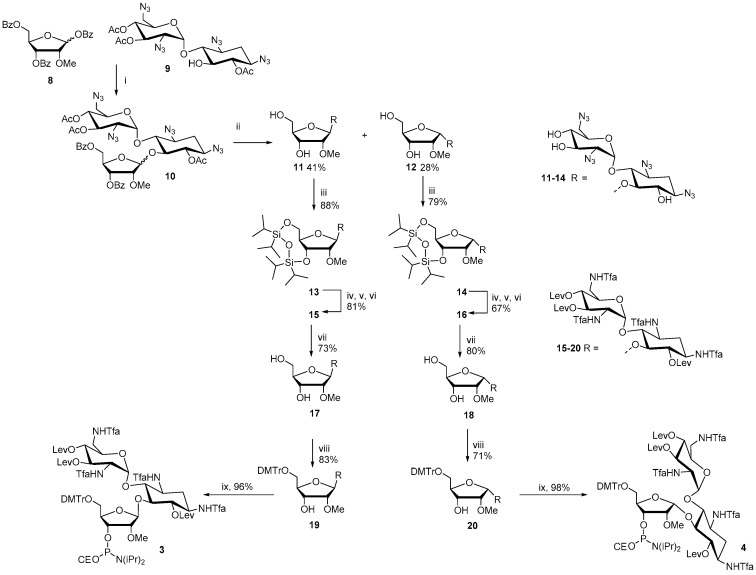
(i) TMSTf, DCM, (ii) NaOMe, MeOH, (iii) ClSi(iPr)_2_OSi(iPr)_2_Cl, Py, (iv) PMe_3_, toluene, aq NH_3_, dioxane, (v) TfaOMe, TEA, MeOH, (vi) Lev_2_O, DMAP, Py, (viii) DMTrCl, Py, (ix) ClP(N*i*Pr_2_)OCE, TEA, DCM.

The synthesis of the ribostamycin-derived phosphoramidites **3** and **4** is outlined in Scheme 1. TMSTf-promoted glycosidation between 1,3,5-di-*O*-benzoyl-2-*O*-methyl-α/β-d-ribofuranose (**8**) and 6,3′,4′-tri-*O*-acetyl-1,3,2′,6′-tetraazido neamine (**9**) [26] gave an anomeric mixture (β:α = 1:2) of peracylated 2′′-*O*-methyl ribostamycin (**10**) in 81% yield. A sodium methoxide-catalyzed transesterification yielded 1,3,2′,6′-tetraazido-2′′-*O*-methyl ribostamycins that could be isolated as the pure β- (**11**) and α-anomer (**12**) by a simple column chromatography. **11** and **12** were then subjected to a treatment with 1,3-dichloro-1,1,3,3-tetraisopropyldisiloxane to protect the 5′′- and 3′′-hydroxyl groups (**13** and **14**). The azide masks of **13** and **14** were reduced by a Staudinger reaction, the exposed amino groups were trifluoroacetylated and the hydroxyl groups were levulinoylated to give fully protected 2′′-*O*-methyl ribostamycins **15** and **16**. The 5′′-*O*, 3′′-*O*-1,1,3,3-tetraisopropyldisiloxane protection was then removed by a treatment of triethylamine trihydrofluoride and the exposed 5′′-hydroxy group was selectively 4′,4′-dimethoxytritylated to give **19** and **20**. The whole protecting group manipulation from **11** to **19** and from **12** to **20** could be carried out in 43% and 30% overall yields, respectively. Phosphitylation of the 3′′-OH group with 2-cyanoethyl *N,N*-diisopropylphosphoramido chloridite gave **3** and **4** in nearly quantitative yields.

### 2.2. Synthesis of Aminoglycoside 2′-O-Methyl Oligonucleotide Fusions Using ***3*** and ***4***

**Figure 2:**
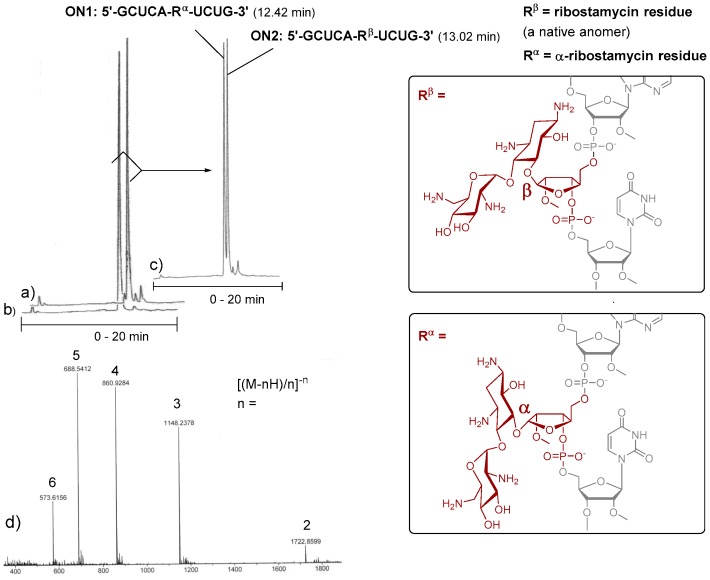
Intra-chain fusions of ribostamycins and a 2′-*O*-methyl oligoribonucleotide (the test syntheses for the evaluation of the coupling efficiency of **3** and **4**). Ion exchange HPLC profiles of crude **ON2** (**a**), of crude **ON1** (**b**) and of a mixture of **ON1** and **ON2** (1:1) (**c**); MS (ESI-TOF) spectrum of **ON2** (**d**); HPLC conditions: An analytical (150 × 5 mm, monolithic) ion exchange column, flow rate 0.5 mL min^−1^, detection at 260 nm, a gradient elution (0–20 min) from 17 mmol L^−1^ to 100 mmol L^−1^ NaClO_4_ in aqueous 20 mmol L^−1^ Tris.

To evaluate the applicability of **3** and **4** in the automated chain assembly, intra-chain fusions of the ribostamycins and a random 2′-*O*-methyl RNA sequence (5′-GCUCA-R-UCUG-3′, **ON1**: R = α-ribostamycin residue (R^α^) and **ON2**: R = ribostamycin residue (R^β^)) were first synthesized (Figure 2). The coupling efficiency was evaluated by DMTr-assay. A double phosphoramidite coupling using 0.1 mol L^−1^ **3** and **4** in acetonitrile, benzylthiotetrazol as an activator and a 600 s coupling time (2 × 600 s), followed by the standard oxidation step, gave ca. 95% coupling yield for both building blocks. Otherwise, the oligonucleotides were assembled using the standard RNA coupling cycle (a 300 s coupling time used for the 2′-*O*-methyl nucleoside building blocks). After the chain assembly, the levulinoyl groups of the ribostamycin moieties were removed on support with a mixture of hydrazinium acetate (NH_2_NH_2_·OH_2_, pyridine, AcOH, 0.124/4/1, *v*/*v*/*v*, 2 × 10 min at 25 °C), and the supports were then subjected to concentrated ammonia (overnight at 55 °C) [17]. The released **ON1** and **ON2** were analysed by ion exchange HPLC. As seen in the HPLC profiles of the crude products (Figure 2a,b), the automated synthesis could be successfully carried out. Interestingly, the chiral integrity of the ribostamycin moieties also affects the retention times of the conjugates in the HPLC profiles (Figure 2c).

**Figure 3:**
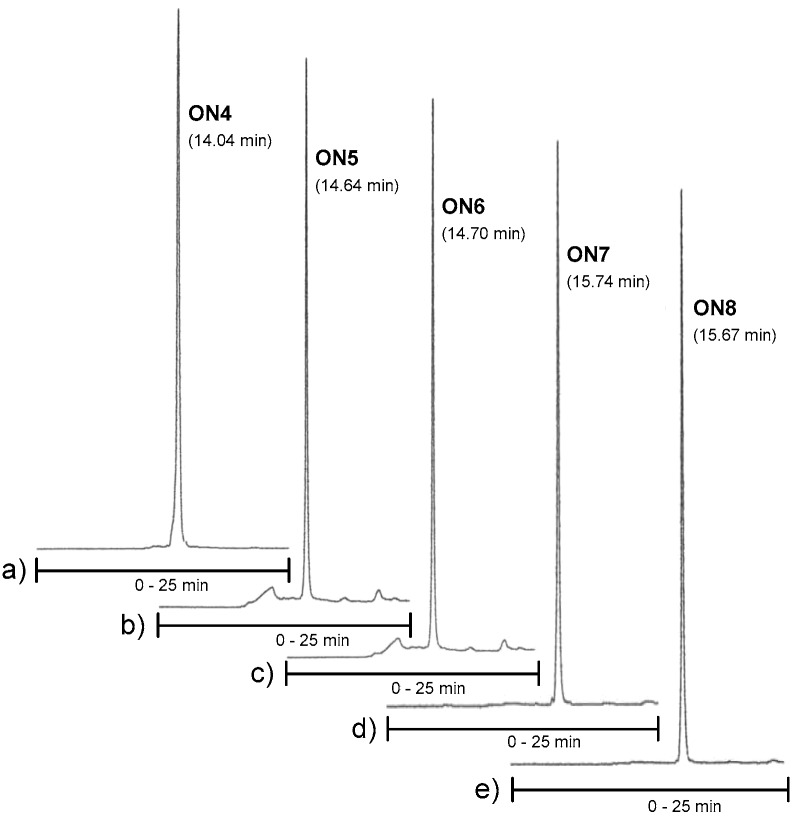
RP HPLC profiles of **ON4**–**ON6**. Conditions: An analytical RP HPLC column (C18, 250 × 5 mm, 5 µm), flow rate: 1.0 mL min^−1^, detection at 260 nm, a gradient elution (0–25 min) from 0 to 50% acetonitrile in 0.1 mol L^−1^ aqueous triethylammonium acetate.

Conjugates **ON4**–**ON6**, which aimed to clamp a purine-rich DNA strand (a sequence of C-Myc promoter 1), were then synthesized on our previously described neomycin-derived LCAA-CPG support (**7**) [22]. A manual coupling of **3** and **4** to support **7** was carried out (see the material and methods) and then the 2′-*O*-methyl oligoribonucleotide chain, including the 2′-deoxy oligonucleotide turn (TCTCT), was assembled in a standard manner. After the chain assembly, the levulinoyl groups of the ribostamycin moieties were removed on support using the hydrazine acetate treatment as mentioned above. The solid-supported conjugates were then exposed to a mixture of NaOMe in methanol (0.1 mol L^−1^, for 2 h at 25 °C, i.e., acetyl removal of the neomycin moieties and cleavage of the succinyl linker), and the deprotection was continued by ammonolysis (overnight at 55 °C, i.e., removal of the Tfa-protections and of the Bz-protections of cytosine bases) [22]. Conjugates **ON7** and **ON8** were synthesized following the procedure described for **ON1** and **ON2**. RP HPLC and MS (ESI-TOF) data of the conjugates **ON1**, **ON2**, **ON4**–**ON8** are shown in Figure 2a,b and Figure 3 (and Table S1), respectively. Isolated yields ranged from 10–26%.

### 2.3. UV-Melting Profile Analysis

**Scheme 2:**
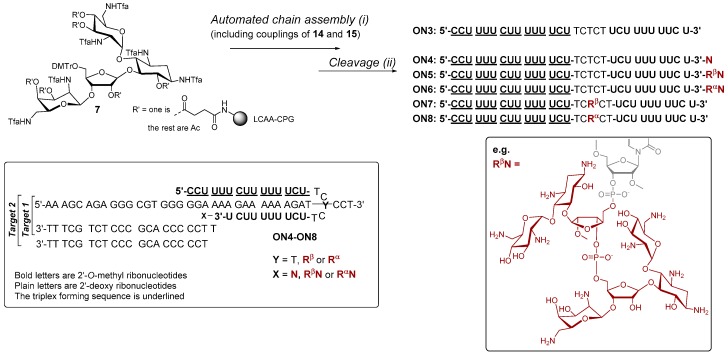
Synthesis of fusions of aminoglycosides and 2′-*O*-methyl 2′-deoxy oligoribonucleotide hybrids (**ON4**–**ON6**). The proposed clamp structures between **ON4**–**ON8** and the purine-rich sequence of C-Myc promoter 1 (*Target 1* and *Target 2*) are described. (i) phosphoramidite coupling cycle using benzylthiotetrazol as an activator; (ii) hydrazinium acetate (NH_2_NH_2_·OH_2_, pyridine, AcOH, 0.124/4/1, *v*/*v*/*v*, 2 × 10 min at 25 °C) (**ON5**–**ON8**), 0.1 mol L^−1^ NaOMe in methanol (2 h at 25 °C) (**ON4**–**ON6**), concentrated ammonia (overnight at 55 °C) (**ON3**–**ON8**).

**Figure 4:**
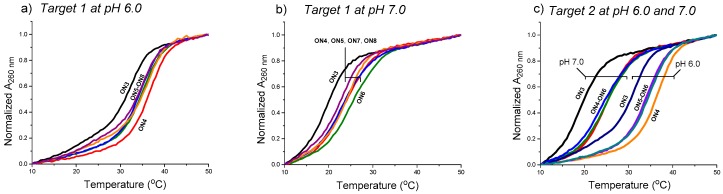
UV-melting profiles of the triple helical constructs (the duplex melting range, *T*_m_ = 65 °C excluded). Conditions: 2.0 each oligonucleotide (*Target 1 or 2* + **ON3**–**ON8**, cf. Figure 3), 10 mmol L^−1^ sodium cacodylate, pH 6.0 (**a**,**c**) and pH 7.0 (**b**,**c**), 0.1 mol L^−1^ NaCl, 0.2 °C/min, detection at 260 nm.

The effect of the aminoglycosides moieties (ribostamycin and neomycin) to stabilize the clamp structures targeted to a purine-rich DNA single strand (a sequence of c-Myc promoter 1) (cf. Scheme 2) has been studied by UV-melting profile experiments (Figure 4 and Table 1). The measurements were carried out using 2 µmol L^−1^ of each oligonucleotide in a mixture of 10 mmol L^−1^ sodium cacodylate and 0.1 mol L^−1^ NaCl at pH 6.0 and 7.0. The temperature was changed at a rate of 0.2 °C min^−1^. In each case, a biphasic melting curve was observed (the duplex melting range, *T*_m_ = 65 °C, excluded in Figure 4) and the *T*_m_^3^-values were extracted from the first inflection point. The *T*_m_^3^-values of the triple helical clamps were expectedly higher (ca. 10 °C) at pH 6.0 than at pH 7.0. As shown, the overhanging 3′-aminoglycoside moiety of the conjugates increased the stability of the clamps in each case. The acidic conditions (pH 6.0 vs. 7.0) did not show a marked role in ∆*T*_m_^3^-values. The 3′-neomycin conjugate (**ON4**) increased the stability of the clamps (Target 1 and Target 2) by ∆*T*_m_^3^ = +4.0–+5.8 °C. The incorporation of the ribostamycin unit to the 3′-aminoglycosides overhang (**ON5** and **ON6**) seemed to elicit, however, slightly decreased ∆*T*_m_^3^-values (e.g., **ON4**: +5.8 °C vs. **ON5**: +4.8 °C and **ON6**: +5.5 °C with Target 2 at pH 7.0). **ON6** did an exception with Target 1 at pH 7.0 (**ON6**: ∆*T*_m_^3^ = +5.1 vs. **ON4**: ∆*T*_m_^3^ = +4.0). A slightly increased stability was also observed, when the ribostamycin was incoporporated into the 2′-deoxy oligoribonucleotide turn (**ON7** and **ON8**: ∆*T*_m_^3^ = +2.6 °C–+4.3 °C).

## 3. Discussion

### 3.1. Synthesis of the Building Blocks ***3*** and ***4*** and of the Aminoglycoside-Oligonucleotide Conjugates ***ON1***, ***ON2***, ***ON4**–**ON8***

Phosphoramidite building blocks 3 and 4 could be synthesized in relatively high yields (Scheme 1). Despite the multistep synthesis, the overall yields of 3 and 4 (calculated from 6,3′,4′-tri-*O*-acetyl-1,3,2′,6′-tetraazido neamine 9 [26]) were 19% and 10%, respectively. The chiral integrity of the anomers was confirmed by a NOESY spectrum that showed a correlation between the H1′′ and 2′-*O*-methoxy groups of the β^1′′−5^-ribostamycins (e.g., 13). Building blocks 3 and 4 could be efficiently incorporated into oligonucleotide sequences using either an automated double phosphoramidite coupling (2 × 600 s coupling time) or a manual coupling (see details in the Materials and Methods section). In the manual coupling, the concentration of the building blocks could be increased to 0.11 mol L^−1^ (after the addition of benzylthiotetrazol) that improved the coupling efficiency (Note: In the synthesizer, the initial concentration of the phosphoramidites is 0.1 mol L^−1^ that is diluted to one half by the solution of benzylthiotetrazol). The *O*-levulinoyl/*N*-trifluoroacetyl (Lev/Tfa)-protecting group combination for 3 and 4 was applied. The reason for this protecting group scheme was the selective on-support removal of the Lev protections (by a hydrazinium acetate treatment in the presence of *N*-Tfa groups) that suppressed plausible *O*→*N* acyl migration. The NaOMe-catalyzed methanolysis was sufficient to selectively remove *O*-acetyl groups from the neomycin moiety (7) and to eliminate *N*-acylated side products (ON4) [22], but the Lev/Tfa-combination is evidently [17] the superior protecting group scheme, when the number of 1,2-aminoethanol-moieties increases. Thus, two-step- (ON1, ON2, ON4, ON7 and ON8) and three-step-treatments (ON5 and ON6) with hydrazinium acetate, NaOMe/MeOH and ammonolysis were used to release the conjugates.

### 3.2. The Effect of the Aminoglycoside Moieties of ***ON1***, ***ON2***, ***ON4**–**ON8*** on the Triple Helical Constructs 

According to UV-melting profiles, the aminoglycoside moieties (ribostamycin and neomycin) increased the stability of the clamp structures in each case (Δ*T*_m_ = +2.2–+5.8 °C), but the effect remained modest. The Watson-Hoogsteen groove (the groove between the pyrimidine strands) of the DNA-triple helix may bind multiple aminoglycosides (neomycin primarily) and the binding has been proposed to be involved in the amino groups of neomycin rings II and IV [27]. The elongated 3′-aminoglycoside overhang of ON5 and ON6 (containing one biosamine and two neamines in the ribose-phosphodiester backbone) probably could not reach the optimal binding contact needed for the groove binding and the stability did not increase compared to 3′-neomycin moiety (ON4: Δ*T*_m_^3^ = +4.0–+5.8 °C). The phosphodiester bond between the neomycin and ribostamycin units may also disturb the binding. The incorporation of the ribostamycin units into the 2′-deoxy oligonucleotide turn increased the stability of the clamps by Δ*T*_m_ = +2.6–+4.3 °C. The stability of the clamps may hence be further increased by incorporation of aminoglycosides at both terminus of the clamp. Further studies may also be needed to evaluate the influence of the longer spacers between the oligonucleotide and the aminoglycoside overhang.

## 4. Materials and Methods

### 4.1. General Remarks

MeCN, pyridine and dichloromethane were dried over 3Å molecular sieves and triethylamine over CaH_2_. NMR spectra were recorded using a 500 MHz instrument. The chemical shifts for ^1^H and ^13^C NMR resonances are given in parts of million from the residual signal of the deuterated solvents (CD_3_OD and CD_3_CN). ^31^P NMR resonance shifts are compared to external H_3_PO_4_. Mass spectra were recorded using electrospray ionization (ESI-TOF).

*1,3,2′,6′-Tetraazido-2′′-O-methyl ribostamycin* (**11** *and* **12**). 6,3′,4′-Tri-*O*-acetyl-1,3,2′,6′-tetraazido neamine (**9**, 1.6 g, 2.9 mmol) [25] and 1,3,5-di-*O*-benzoyl-2-*O*-methyl-α/β-d-ribofuranose (**8**, 3.4 g, 7.1 mmol) were dissolved in dichloromethane (20 mL) and the mixture was cooled down to 0 °C. Trimethylsilyl trifluoromethanesulfonate (0.58 mL, 0.32 mmol) was slowly added to the mixture and the reaction was stirred at 0 °C for 2 h and then at room temperature for 2 h under a nitrogen atmosphere. Dichloromethane (50 mL) and saturated NaHCO_3_ (20 mL) were added to the mixture. The organic layer was separated, washed with saturated NaCl, dried over Na_2_SO_4_, filtered and evaporated to dryness. The residue was purified by silica gel chromatography (30% EtOAc in petroleum ether) to give 2.13 g (81%) of **10** as colourless oil (anomeric mixture, β:α = 1:2). The peracylated ribostamycine (**10**) was dissolved in 0.1 mol L^−1^ methanolic sodium methoxide (5.0 mL). The mixture was stirred at ambient temperature for 1 h, neutralized by addition of strongly acidic cation-exchange resin, and filtered. The filtrate was evaporated to dryness and purified by silica gel chromatography (1. EtOAc, 2. 10% MeOH in CH_2_Cl_2_) to give 0.55 g (41%) of **11** (β anomer) and 0.37 g (28%) of **12** (α anomer). **11**: ^1^H NMR (500 MHz, CD_3_OH): δ 5.89 (d, 1H, *J* = 3.8 Hz), 5.45 (b, 1H), 4.21–4.17 (m, 2H), 3.92–3.89 (m, 2H), 3.82–3.79 (m, 2H), 3.70–3.63 (m, 3H), 3.58–3.52/m, 2H), 3.54 (s, 3H), 3.48–3.42 (m, 3H), 3.39 (m, 1H), 3.13 (dd, 1H, *J* = 10.6 Hz & 3.8 Hz), 2.24 (m, 1H), 1.40 (m, 1H); ^13^C (125 MHz, CD_3_OH): δ 105.5, 96.7, 84.6, 84.0, 83.1, 76.0, 75.6, 71.8, 71.2, 70.7, 69.8, 63.1, 62.2, 60.6, 59.9, 57.3, 51.2, 31.6; HRMS (ESI-TOF) *m*/*z*: [M + Na]^+^ calcd. for C_18_H_28_N_12_NaO_10_ 595.1949, found 595.1930. **12**: ^1^H NMR (500 MHz, CD_3_OH): δ 5.84 (d, 1H, *J* = 3.9 Hz), 5.54 (d, 1H, *J* = 4.6 Hz), 4.27–4.24 (m, 3H), 3.87 (dd, 1H, *J* = 5.0 Hz, both), 3.81 (dd, 1H, *J* = 10.0 Hz & 9.1 Hz), 3.70–3.61 (m, 4H), 3.55 (s, 3H), 3.55–3.43 (m, 5H), 3.38 (m, 1H), 3.24 (dd, 1H, *J* = 10.5 Hz & 4.0 Hz), 2.25 (ddd, 1H, *J* = 12.9 Hz, 4.4 Hz & 4.4 Hz), 1.44 (ddd, 1H, *J* = 12.3 Hz, each); ^13^C (125 MHz, CD_3_OH): δ 103.0, 97.4, 86.6, 84.6, 81.1, 77.8, 74.9, 71.8, 71.5, 71.1, 68.3, 64.0, 61.8, 60.1, 59.5, 57.6, 51.1, 31.6; HRMS (ESI-TOF) *m*/*z*: [M + Na]^+^ calcd. for C_18_H_28_N_12_NaO_10_ 595.1949, found 595.1929.

*1,3,2′,6′-Tetraazido-3′′,5′′-O-(tetraisopropyldisiloxane-1,3-diyl)-2′′-O-methyl ribostamycin (***13***).* 1,3-Dichloro-1,1,3,3-tetraisopropyldisiloxane (350 μL, 1.1 mmol) was added to a mixture of **11** (0.55 g, 0.96 mmol) in pyridine (3.0 mL). The mixture was stirred overnight at ambient temperature and then dichloromethane and saturated NaHCO_3_ were added. The organic layer was separated, washed with saturated NaCl, dried over Na_2_SO_4_, filtered and evaporated to dryness. The residue was purified by silica gel chromatography (5% MeOH in CH_2_Cl_2_) to yield 0.69g (88%) of the product (**13**) as white foam. ^1^H NMR (500 MHz, CD_3_OH): δ 6.10 (d, 1H, *J* = 3.8 Hz), 5.27 (s, 1H), 4.53 (dd, 1H, *J* = 9.0 Hz & 4.2 Hz), 4.20 (m, 1H), 4.12 (dd, 1H, *J* = 12.9 Hz & 1.9 Hz), 3.96 (dd, 1H, *J* = 12.8 Hz & 2.5 Hz), 3.92–3.86 (m, 3H), 3.67–3.62 (m, 2H), 3.59 (s, 3H), 3.57–3.41 (m, 4H), 3.31–3.24 (m, 2H), 3.07 (dd, 1H, *J* = 10.5 Hz & 3.8 Hz), 2.24 (m, 1H), 1.36 (ddd, *J* = 12.5 Hz, each), 1.16–1.06 (m, 28H); ^13^C (125 MHz, CD_3_OH): δ 107.8, 96.3, 85.9, 84.8, 80.4, 76.7, 75.2, 71.52, 71.50, 71.1, 70.3, 63.5, 60.9, 60.6, 60.1, 51.5, 31.8, 16.8, 16.62, 16.59, 16.5, 16.40, 16.37, 16.31, 16.29, 16.24, 16.16, 16.1, 13.5, 13.4, 13.1, 12.9, 12.5, 12.4; HRMS (ESI-TOF) *m*/*z*: [M + K]^+^ calcd. for C_30_H_54_KN_12_O_11_Si_2_ 853.3211, found 853.3148.

*1,3,2′,6′-Tetraazido-3′′,5′′-O-(tetraisopropyldisiloxane-1,3-diyl)-2′′-O-methyl-α^1′′−5^-ribostamycin (***14***).* The α-anomer (**14**) was synthesized as described for **13** above. 0.37 g (0.64 mmol) of **12** gave 0.42 g (79%) of **14** as white foam. ^1^H NMR (500 MHz, CD_3_OH): δ 5.67 (d, 1H, *J* = 3.8 Hz), 5.43 (d, 1H, *J* = 4.1 Hz), 4.40 (dd, 1H, *J* = 7.7 Hz & 5.1 Hz), 4.28 (m, 1H), 4.20 (m, 1H), 4.04–4.00 (m, 2H), 3.96 (dd, 1H, *J* = 12.8 Hz & 4.4 Hz), 3.87 (dd, 1H, *J* = 10.3 Hz & 9.1 Hz), 3.66 (s, 3H), 3.64–3.61 (m, 2H), 3.56–3.43 (m, 5H), 3.40 (dd, 1H, *J* = 9.6 Hz & 9.2 Hz), 3.24 (dd, 1H, *J* = 10.5 Hz & 3.9 Hz), 2.24 (m, 1H), 1.46 (ddd, 1H, *J* = 12.3 Hz, each), 1.16–1.06 (m, 28H); ^13^C (125 MHz, CD_3_OH): δ 103.4, 97.9, 87.3, 81.4, 80.0, 77.7, 74.9, 72.0, 71.3, 71.14, 71.08, 63.4, 61.0, 59.9, 59.22, 59.18, 51.1, 31.5, 16.5, 16.42, 16.38, 16.30, 16.29, 16.2, 16.1; HRMS (ESI-TOF) *m*/*z*: [M + K]^+^ calcd. for C_30_H_54_KN_12_O_11_Si_2_ 853.3211, found 853.3174.

*6,3′,4′-Tri-O-levulinoyl-3′′,5′′-O-(tetraisopropyldisiloxane-1,3-diyl)-tetra-N^1^,N^3^,N^2′^,N^6′^-trifluoroacetyl-2′′-O-methyl ribostamycin* (**15**). Trimethylphosphine (1 mol L^−1^ Me_3_P in toluene, 5.1 mL, 5.1 mmol) was added to a mixture of **13** (0.42 g, 0.51 mmol) in water–dioxane (1:4, *v*/*v*, 5.0 mL). The mixture was stirred at ambient temperature for 4 h under nitrogen and then concentrated ammonia (1.0 mL) was added. After overnight reaction, the mixture was evaporated to dryness and the residue was coevaporated with pyridine. The residue was dissolved in methanol (4.0 mL) and triethylamine (2.0 mL), and methyl trifluoroacetate (0.41 mL, 4.1 mmol) was added to the mixture. The reaction was stirred at ambient temperature for 5 h. Saturated NaHCO_3_ was added and the mixture was extracted with ethyl acetate. The combined organic layers were dried over Na_2_SO_4_, filtered and evaporated to dryness. The residue was dissolved in pyridine (5.0 mL) and then freshly prepared levulinic anhydride (0.66 g, 3.1 mmol) and a catalytic amount of DMAP were added to the mixture. After overnight stirring, methanol and saturated NaHCO_3_-were added to the mixture and the crude product was extracted with ethyl acetate. The combined organic layers were dried over NaSO_4_, filtered and evaporated to dryness. The residue was subjected to a silica gel chromatography (30% petroleum ether in ethyl acetate) to yield 0.58 g (81 %) of **15** as white foam. ^1^H NMR (500 MHz, CD_3_OH): δ 6.05 (d, 1H, *J* = 3.7 Hz), 5.51 (dd, 1H, *J* = 10.1 Hz, both), 4.97–4.90 (m, 3H), 4.37 (d, 1H, *J* = 3.9 Hz), 4.34 (m, 1H), 4.26–4.11 (m, 2H), 4.08–4.03 (m, 2H), 3.95 (dd, 1H, *J* = 13.2 Hz & 1.5 Hz), 3.84–3.80 (m, 2H), 3.73 (m, 1H), 3.64 (dd, 1H, *J* = 14.6 Hz & 3.0 Hz), 3.67 (s, 3H), 3.59–3.43 (m, 2H), 2.83–2.41 (m, 12H), 2.17, 2.16 and 2.15 (3 × s, 9H), 2.04–1.95 (m, 2H), 1.14–1.06 (m, 28H); ^13^C (125 MHz, CD_3_OH): δ 206.4, 205, 9, 205.0, 170.9, 170, 8, 170.4, 156.4–155.6 (m), 118.0–111.0 (m), 107.6, 93.8, 82.9, 82.4, 78.7, 73.9, 73.8, 69.0, 68.7, 67.8, 65.4, 58.3, 56.6, 49.9, 49.2, 47.2, 46.7, 37.9, 35.7, 35.6, 35.5, 29.5, 26.7, 26.65, 26.62, 26.57, 26.1, 25.9, 25.8, 25.7, 15.02, 14.93, 14.92, 14.89, 14.88, 14.78, 14.76, 14.7, 14.6, 14.5, 11.9, 11.6, 11.5, 11.0, 10.8; HRMS (ESI-TOF) *m*/*z*: [M + Na]^+^ calcd. for C_53_H_76_F_12_N_4_NaO_21_Si_2_ 1411,4247, found 1411.4283.

*6,3′,4′-Tri-O-levulinoyl-3′′,5′′-O-(tetraisopropyldisiloxane-1,3-diyl)-tetra-N^1^,N^3^,N^2′^,N^6′^-trifluoroacetyl-2′′-O-methyl-α^1′′−5^-ribostamycin* (**16**). The α-anomer (**16**) was synthesized as described for **15** above. 0.41 g (0.50 mmol) of **14** gave 0.42 g (59%) of **16** as white foam. ^1^H NMR (500 MHz, CD_3_OH): δ 6.02 (d, 1H, *J* = 3.9 Hz), 5.24 (dd, 1H, *J* = 10.7 Hz & 9.6 Hz), 5.06 (dd, 1H, *J* = 10.1 Hz & 9.1 Hz), 5.02 (d, 1H, *J* = 3.9 Hz), 4.96 (dd, 1H, *J* = 9.9 Hz, both), 4.39 (dd, 1H, *J* = 10.9 Hz & 3.9 Hz), 4.25–4.19 (m, 2H), 4.13 (m, 1H), 4.04–4.00 (m, 2H), 3.97–3.91 (m, 2H), 3.88 (dd, 1H, *J* = 12.7 Hz & 2.8 Hz), 3.80 (dd, 1H, *J* = 12.7 Hz & 4.3 Hz), 3.66–3.58 (m, 2H), 3.54 (s, 3H), 3.55–3.50 (m, 1H), 2.85–2.75 (m, 6H), 2.71–2.40 (m, 6H), 2.17 (s, 3H), 2.15 (s, 2 × 3H), 2.04–1.95 (m, 2H), 1.14–1.05 (m, 28H); ^13^C (125 MHz, CD_3_OH): δ 207.8, 207.4, 207.2, 172.6, 172.02, 171.98, 158.4–156.8 (m), 119.5–111.5 (m), 102.9, 95.7, 85.3, 81.5, 79.7, 76.1, 74.4, 70.8, 70.2, 68.5, 67.5, 60.5, 58.5, 51.8, 48.8, 48.5, 38.6, 37.2, 37.0, 30.9, 28.3, 28.1, 27.8, 27.5, 27.2, 16.5, 16.4, 16.3, 16.24, 16.21, 16.19, 16.1, 13.3, 13.1, 13.0, 12.6, 12.5; HRMS (ESI-TOF) *m*/*z*: [M + Na]^+^ calcd. for C_53_H_76_F_12_N_4_NaO_21_Si_2_ 1411,4247, found 1411.4190.

*6,3′,4′-Tri-O-levulinoyl-tetra-N^1^,N^3^,N^2′^,N^6′^-trifluoroacetyl-2′′-O-methyl ribostamycin* (**17**). Compound **15** (0.48 g, 0.35 mmol) was dissolved in a mixture of triethylamine trihydrofluoride and acetonitrile (1:10, *v*/*v*, 2.0 mL). The reaction was stirred overnight at ambient temperature, 10% aqueous KH_2_PO_4_ was added and the mixture was extracted with ethyl acetate. The combined organic layers were washed with saturated NaHCO_3_ and saturated NaCl, dried with Na_2_SO_4_, filtered and evaporated to dryness. The crude product was purified by a silica gel chromatography (10 % MeOH in CH_2_Cl_2_) to yield 0.35 g (73 %) of **17** as white foam. ^1^H NMR (500 MHz, CD_3_OH): δ 6.11 (d, 1H, *J* = 3.5 Hz), 5.21 (dd, 1H, *J* = 10.6 Hz & 9.6 Hz), 5.16 (d, 1H, *J* = 4.3 Hz), 4.99 (dd, 1H, *J* = 10.2 Hz & 9.6 Hz), 4.95 (dd, 1H, *J* = 9.8 Hz, both), 4.38 (dd, 1H, *J* = 10.8 Hz & 3.9 Hz), 4.24 (m, 1H), 4.15 (m, 1H), 4.12 (dd, 1H, *J* = 4.1 Hz, both), 4.04–4.01 (m, 2H), 3.85 (dd, 1H, *J* = 10.0 Hz & 8.8 Hz), 3.78 (m, 1H), 3.65 (dd, 1H, *J* = 14.4 Hz & 4.0 Hz), 3.56–3.52 (m, 3H), 3.47 (dd, 1H, *J* = 14.5 Hz & 2.4 Hz), 3.39 (s, 3H), 2.84–2.72 (m, 6H), 2.70–2.41 (m, 6H), 2.19 (s, 3H), 2.17 (s, 3H), 2.14 (s, 3H), 2.01–1.97 (m, 2H); ^13^C (125 MHz, CD_3_OH): δ 208.0, 207.7, 207.6, 172.6, 171.98, 171.96, 158.5–156.9 (m), 119.5–112.5 (m), 108.1, 95.1, 84.7, 85.6, 82.9, 75.5, 75.1, 70.1, 69.0, 68.6, 67.5, 61.6, 57.5, 48.7, 48.5, 39.0, 37.3, 37.1, 30.8, 28.3, 28.2, 27.8, 27.5; HRMS (ESI-TOF) *m*/*z*: [M + Na]^+^ calcd. for C_41_H_50_F_12_N_4_NaO_20_ 1169.2724, found 1169.2744.

*6,3′,4′-Tri-O-levulinoyl-tetra-N^1^,N^3^,N^2′^,N^6′^-trifluoroacetyl-2′′-O-methyl-α^(1′′−5)^-ribostamycin* (**18**). The α-anomer (**18**) was synthesized as described for **17** above. 0.35 g (0.25 mmol) of **16** gave 0.28 g (80%) of **18** as white foam. ^1^H NMR (500 MHz, CD_3_OH): δ 6.09 (d, 1H, *J* = 2.6 Hz), 5.22 (dd, 1H, *J* = 10.7 Hz & 9.6 Hz), 5.08–5.04 (m, 2H), 4.94 (dd, 1H, *J* = 9.9 Hz & 9.8 Hz), 4.36 (dd, 1H, *J* = 10.9 Hz & 3.9 Hz), 4.22 (m, 1H), 4.17–4.12 (m, 2H), 4.04–3.98 (m, 3H), 3.89 (dd, 1H, *J* = 10.1 Hz & 8.8 Hz), 3.65 (dd, 1H, *J* = 14.8 Hz & 3.5 Hz), 3.58–3.54 (m, 3H), 3.49 (dd, 1H, *J* = 14.4 Hz & 2.0 Hz), 3.35 (s, 3H), 2.86–2.41 (m, 12H), 2.17 (s, 2 × 3H), 2.15 (s, 3H), 2.04–1.94 (m, 2H); ^13^C (125 MHz, CD_3_OH): δ 207.9, 207.8, 207.5, 172.9, 172.0, 158.4–156.9 (m), 119.4–112.5 (m), 103.8, 96.1, 86.9, 84.1, 80.9, 77.2, 74.3, 70.2, 68.5, 67.7, 67.6, 61.8, 57.2, 53.5, 48.7, 48.6, 38.6, 37.3, 37.2, 37.1, 30.9, 28.4, 28.3, 28.0, 27.5; HRMS (ESI-TOF) *m*/*z*: [M + Na]^+^ calcd. for C_41_H_50_F_12_N_4_NaO_20_ 1169.2724, found 1169.2716.

*5′′-O-(4,4′-Dimethoxytrityl)-6,3′,4′-tri-O-levulinoyl-tetra-N^1^,N^3^,N^2′^,N^6′^-trifluoroacetyl-2′′-O-methyl ribostamycin* (**19**). 17 (0.35 g, 0.31 mmol) was dissolved in dry pyridine (2.0 mL) and 4′,4′-dimethoxytritylchloride (0.12 g, 0.34 mmol) was added. The mixture was stirred overnight at ambient temperature, poured to saturated aqueous NaHCO_3_ and the product was extracted with dichloromethane. The organic layers were combined, dried over Na_2_SO_4_, filtered and evaporated to dryness. The residue was purified by silica gel chromatography (10% MeOH in CH_2_Cl_2_) to give 0.37 g (83%) of the product (**19**) as white foam. ^1^H NMR (500 MHz, CD_3_OH): δ 7.48 (2H, d, *J* = 7.5 Hz), 7.35–7.32 (m, 6H), 7.25 (m, 1H), 6.92 (d, 4H, *J* = 8.8 Hz), 6.16 (d, 1H, *J* = 4.0 Hz), 5.18 (d, 1H, *J* = 4.9 Hz), 5.14 (dd, 1H, *J* = 10.4 Hz & 9.8 Hz), 5.07 (dd, 1H, *J* = 10.2 Hz & 9.7 Hz), 4.88 (dd, 1H, *J* = 9.9 Hz, both), 4.23 (m, 1H), 4.16 (m, 1H), 4.05–3.97 (m, 4H), 3.90 (dd, 1H, *J* = 10.2 Hz & 8.9 Hz), 3.85 (m, 1H), 3.82 (s, 2 × 3H), 3.63 (dd, 1H, *J* = 4.6 Hz, both), 3.54 (dd, 1H, *J* = 14.6 Hz & 4.3 Hz), 3.42 (dd, 1H, *J* = 14.6 Hz & 3.4 Hz), 3.31 (s, 3H), 3.21 (d, 2H, *J* = 4.0 Hz), 2.84–2.71 (m, 6H), 2.69–2.40 (m, 6H), 2,17 (s, 3H), 2.16 (s, 3H), 2.14 (s, 3H), 2.07 (ddd, 1H, *J* = 12.8 Hz, each), 2.00 (ddd, 1H, *J* = 12.8 Hz, 4.6 Hz & 4.6 Hz); ^13^C (125 MHz, CD_3_OH): δ 207.9, 207.6, 207.4, 172.6, 172.2, 171.8, 158.84, 158.80, 158.4–156.6 (m), 145.1, 135.7, 135.4, 130.2, 130.1, 128.0, 127.4, 126.4, 119.5–112.5 (m), 112.8, 112.7, 108.3, 94.9, 86.2, 84.8, 83.9, 83.2, 75.1, 75.0, 70.2, 69.2, 69.0, 66.9, 62.5, 57.3, 54.4, 51.2, 48.7, 48.5, 39.1, 37.3, 37.1, 30.8, 28.2, 28.15, 28.12, 27.9, 27.49, 27.46. HRMS (ESI-TOF) *m*/*z*: [M + Na]^+^ calcd. for C_62_H_68_F_12_N_4_NaO_22_ 1471.4031, found 1471.4055.

*5′′-O-(4,4′-Dimethoxytrityl)-6,3′,4′-tri-O-levulinoyl-tetra-N^1^,N^3^,N^2′^,N^6′^-trifluoroacetyl-2′′-O-methyl-α^1′′−5^-ribostamycin* (**20**). The α-anomer (**20**) was synthesized as described for **19** above. 0.28 g (0.24 mmol) of **18** gave 0.25 g (71%) of **20** as white foam. ^1^H NMR (500 MHz, CD_3_OH): δ 7.41 (d, 2H, *J* = 7.6 Hz), 7.31–7.28 (m, 6H), 7.22 (m, 1H), 6.86 (d, 4H, *J* = 8.7 Hz), 6.12 (b, 1H), 5.26 (dd, 1H, *J* = 10.4 Hz & 9.9 Hz), 5.16 (d, 1H, *J* = 4.3 Hz), 5.06 (dd, 1H, *J* = 9.9 Hz & 9.4 Hz), 4.95 (dd, 1H, *J* = 9.9Hz, both), 4.38 (dd, 1H, *J* = 10.9 Hz & 3.9 Hz), 4.27–4.14 (m, 3H), 4.08–4.04 (m, 3H), 3.91 (dd, 1H, *J* = 10.2 Hz & 9.1 Hz), 3.81 (m, 1H), 3.79 (s, 2 × 3H), 3.68 (dd, 1H, *J* = 14.8 Hz & 3.2 Hz), 3.50 (dd, 1H, *J* = 14.8 Hz & 2.0 Hz), 3.37 (s, 3H), 3.21 (dd, 1H, *J* = 10.5 Hz & 3.5 Hz), 3.09 (dd, 1H, *J* = 10.4 Hz & 3.2 Hz), 2.84–2.71 (m, 4H), 2.61–2.41 (m, 8H), 2.14 (s, 3H), 2.12 (s, 3H), 2.02 (s, 3H), 2.00–1.96 (m, 2H); ^13^C (125 MHz, CD_3_OH): δ 207.8, 207.4, 207.1, 172.7, 172.9, 171.9, 158.1, 158.4–156.8 (m), 144.9, 135.7, 135.6, 129.9, 127.8, 127.4, 126.5, 119.5–112.5 (m), 112.7, 104.0, 96.2, 86.1, 85.8, 81.1, 77.4, 74.3, 70.2, 68.6, 67.8, 67.7, 63.6, 57.2, 54.3, 51.7, 48.73, 48.70, 38.6, 37.13, 37.05, 31.0, 28.4, 28.17, 28.15, 28.0, 27.5; HRMS (ESI-TOF) *m*/*z*: [M + Na]^+^ calcd. for C_62_H_68_F_12_N_4_NaO_22_ 1471.4031, found 1471.3997.

*2-Cyanoethyl [5′′-O-(4,4′-dimethoxytrityl)-6,3′,4′-tri-O-levulinoyl-tetra-N^1^,N^3^,N^2′^,N^6′^-trifluoroacetyl-2′′-O-methyl ribostamycin-3′′-O-yl]* *N,N-diisopropylphosphoramidite* (**3**). Triethylamine (0.16 mL, 1.3 mmol) and 2-cyanoethyl *N,N*-diisopropylphosphoramidochloridite (0.12 g, 0.52 mmol) were added to a mixture of **19** (0.37 g, 0.26 mmol) in dry dichloromethane (1.0 mL). The mixture was stirred at ambient temperature for 1 h under nitrogen and filtered through a short silica gel column (5% Et_3_N in EtOAc). The product fractions were evaporated to dryness to give 0.40 g (96%) of the product (**3**) as white foam. ^1^H NMR (500 MHz, CD_3_OH): δ 8.00 (d, 1H, *J* = 8.7 Hz), 7.48–7.44 (2H), 7.38–7.27 (m, 6H), 7.20–7.25 (m, 2H), 6.95–6.92 (m, 4H), 6.13 and 6.03 (2 × d, 1H, *J* = 3.8 Hz and 3.9 Hz), 5.15 (d, 1H, *J* = 6.2 Hz), 5.12–5.08 (m, 1H), 5.01–4.97 (m, 1H), 4.92–4.83 (m, 1H), 4.35–3.05 (m, 13H), 3.82 (s, 6H), 3.26 and 3.23 (2 × s, 3H), 2.81–2.31 (m, 14H), 2.13, 2.11 and 2.10 (3 × s, 9H), 2.15–1.96 (m, 2H), 1.32–1.08 (m, 12H); ^13^C (125 MHz, CD_3_OH): δ 207.01, 206.99, 206.45, 206.42, 206.1, 172.9, 172.8, 172.3, 172.2, 171.91, 171.88, 158.8, 157.4–156.6 (m), 145.2, 145.0, 135.61, 135.59, 135.43, 135.37, 130.31, 130.28, 128.1, 127.9, 126.94, 126.90, 118.6, 118.2, 119.5–112.5 (m), 113.1, 113.0, 108.4, 108.3, 94.5, 94.4, 86.5, 86.3, 84.3, 84.2, 83.8, 83.7, 83.3, 83.2, 75.0, 74.8, 74.7, 71.9, 71.8, 70.9, 70.8, 70.1, 70.0, 68.9, 68.8, 67.2, 67.0, 63.1, 62.9, 58.9, 58.8, 58.2, 58.1, 58.0, 57.9, 57.8, 57.6, 57.5, 55.0, 51.6, 51.5, 48.9, 48.8, 48.7, 48.6, 43.2, 43.1, 42.9, 42.8, 39.5, 39.4, 37.6, 37.5, 37.3, 37.2, 31.0, 28.9, 28.8, 28.8, 28.7, 28.0, 27.6, 24.1, 24.0, 23.9, 23.9, 23.8, 23,7, 20.0, 19.9, 19.9, 19.8; ^31^P (200 MHz, CD_3_CN): δ 150.4, 148.9; HRMS (ESI-TOF) *m*/*z*: [M + Na]^+^ calcd. for C_71_H_86_F_12_N_6_O_23_P 1649.5290, found 1649.5232.

*2-Cyanoethyl [5′′-O-(4,4′-dimethoxytrityl)-6,3′,4′-tri-O-levulinoyl-tetra-N^1^,N^3^,N^2′^,N^6′^-trifluoroacetyl-2′′-O-methyl-α^1′′−5^-ribostamycin-3′′-O-yl] N,N-diisopropylphosphoramidite* (**4**). The α-anomer (**4**) was synthesized as described for **3** above. 0.25 g (0.17 mmol) of **20** gave 0.28 g (98%) of **4** as white foam. ^1^H NMR (500 MHz, CD_3_OH): δ 8.00–7.90 (m, 1H), 7.55–7.37 (m, 4H), 7.37–7.16 (m, 7H), 6.91–6.87 (m, 4H), 6.18 and 6.87 (2 × b, 1H), 5.30–4.94 (m, 4H), 4.45–3.00 (m, 13H), 3.80 and 3.79 (2 × s, 6H), 3.38 and 3.28 (2 × s, 3H), 2.80–2.28 (m, 14H), 2.08–1.88 (m, 2H), 2.11, 2.10, 2.09, 2.02, 2.00 and 1.99 (6 × s, 9H), 1.33–1.14 (m, 12H); ^13^C (125 MHz, CD_3_OH): δ 207.0, 206.9, 206.5, 206.2, 206.0, 172.8, 172.6, 172.5, 172.4, 172.1, 172.0, 158.7, 157.5–156.4 (m), 145.2, 145.0, 136.0, 135.7, 135.7, 135.5, 130.1, 130.0, 129.9, 129.9, 127.9, 127.9, 127.8, 127.8, 126.9, 119.4, 118.4, 116.9–110.4 (m), 113.1, 113.1, 113.0, 104.3, 104.0, 96.2, 95.8, 86.1, 85.9, 84.7, 81.8, 80.9, 74.2, 73.8, 70.0, 69.7, 68.9, 68.3, 68.3, 67.8, 63.6, 63.1, 60.0, 59.1, 58.6, 57.7, 57.5, 57.5, 57.4, 54.9, 54.9, 52.0, 51.7, 49.6, 49.2, 48.7, 48.7, 43.2, 42.8, 39.2, 38.7, 37.4, 37.3, 37.3, 37.2, 31.1, 31.0, 28.8, 28.7, 28.2, 28.0, 27.6, 24.0, 23.9, 23.8, 23.7, 20.2, 20.1, 19.8, 19.7; ^31^P (200 MHz, CD_3_CN): δ 150.95, 149.60; HRMS (ESI-TOF) *m*/*z*: [M + Na]^+^ calcd. for C_71_H_86_F_12_N_6_O_23_P 1649.5290, found 1649.5221.

### 4.2. Synthesis of Oligonucleotides ***ON1***, ***ON2***, ***ON4**–**ON8***

Oligonucleotides **ON1**–**ON8** were synthesized on a 0.5 or 1.0 µmol scale using an automatic DNA/RNA synthesizer (Applied Biosystems 3400 DNA synthesizer, Waltham, MA, USA). Benzylthiotetrazol was used as an activator. The chain assembly with commercially available nucleoside phosphoramidites (Glen Research, Manchester, CA, USA) was carried out as usual (with a 300 s coupling time). For the coupling of **3** and **4**, a double coupling procedure with an extended 600 s coupling time was used. According to DMTr-cation assay, **3** and **4** could be incorporated into oligonucleotide chains (**ON1**, **ON2**, **ON7** and **ON8**) with ca. 95% coupling yield. For the synthesis of **ON5** and **ON6**, a manual coupling of **3** and **4** to the neomycin-derived LCAA-CPG-support (**7**) was, however, applied: A solution of **3** and **4** (0.20 mol L^−1^ in acetonitrile, 100 µL, 20 µmol) was mixed with a solution of benzylthiotetrazol (BnSTet, 0.25 mol L^−1^, in dry acetonitrile, 80 µL, 20 µmol). The mixture was suspended with support **7** (L = 10 µmol g^−1^, 50 mg, 0.5 µmol) and the suspension was mixed for 10 min under nitrogen at ambient temperature. The suspension was loaded to a DNA-synthesis column, washed with acetonitrile and then a mixture of acetic anhydride, lutidine and *N*-methyl imidazol in THF (5:5:8:82, *v*/*v*/*v*/*v*) was introduced to the column (i.e., the capping step). After 5 min incubation time, the capping solution was washed away and then a mixture of 0.02 mol L^−1^ I_2_ in pyridine and H_2_O in THF (1:21:213, *v*/*v*/*v*) was introduced to the column (i.e., the oxidation step). After 1 min incubation, the iodine solution was removed, the support was washed with acetonitrile, dried, and the column was then coupled to the DNA/RNA synthesizer. According to DMTr-cation assay ca. 90% coupling yield for **3** and **4** was obtained. The RNA chain assembly with these supports were then carried out as usual. After the chain assembly, the solid supported oligonucleotides (**ON1**, **ON2**, **ON5**–**ON8**) bearing **3** and **4** were treated with a mixture of hydrazine acetate to selectively remove the levulinoyl protections: The synthesis column was removed from the synthesizer, a solution of hydrazinium acetate (NH_2_NH_2_·OH_2_, pyridine, AcOH, 0.124/4/1, *v*/*v*/*v*, 2 × 10 min at 25 °C) was manually introduced to the column, and then the support was washed with pyridine, acetonitrile and dichloromethane and dried. NaOMe-catalyzed transesterification was also used to selectively remove the acetyl groups of the neomycin moieties of **ON4**–**ON6**: The dried supports were removed from the synthesis columns, placed into micro centrifuge tubes and suspended in a mixture of 0.1 mol L^−1^ NaOMe in methanol (1.0 mL). The mixtures were mixed for 2 h at ambient temperature and then 1.0 mol L^−1^ aqueous NH_4_Cl (0.11 mL) was added. The supports were removed by filtration and the filtrates were evaporated to dryness. The partially deprotected residues of **ON4**–**ON6** and the solid-supported **ON1**, **ON2**, **ON7** and **ON8** were finally subjected to concentrated ammonia (overnight at 55°) to completely deprotect the conjugates. The conjugates were purified by HPLC (Figure 2a,b and Figure 3) and their authenticity was verified by MS (ESI-TOF) spectroscopy (Table S1). The isolated yield of the conjugates ranged from 10% to 26%.

### 4.3. UV-Melting Temperature Studies

The melting curves (absorbance vs. temperature) were measured at 260 nm on a PerkinElmer Lambda 35 UV-Vis spectrometer equipped with a multiple cell holder and a Peltier temperature controller. An internal thermometer was also used. The temperature was changed at a rate of 0.2 °C min^−1^. Each *T*_m_^3^-value was determined to be the maximum of the first derivative of the melting curve.

## 5. Conclusions

In this primarily synthetic study, synthesis of phosphoramidite building blocks of 2′′-*O*-methyl ribostamycins **3** and **4** that may be incorporated at any position of the oligonucleotide sequence, have been described. According to DMTr-assay and HPLC analysis of the released conjugates, the building blocks (**3** and **4**) could be efficiently incorporated into oligonucleotide sequences using a double phosphoramidite coupling (2 × 600 s) and using benzylthiotetrazol as an activator. **3** and **4** and a neomycin-derived solid support (**7**) were used for the preparation of aminoglycoside conjugates of 2′-*O*-methyl and 2′-deoxy oligoribonucleotide hybrids that were aimed to clamp a purine-rich DNA single strand (a sequence of c-Myc promoter 1). The potential of the intrachain ribostamycin and 3′-multiaminoglycoside overhangs to act as groove binders to stabilize triple helical region of the DNA-2′-*O*-methyl RNA clamps was demonstrated. According to UV-melting profile analysis, slightly increased clamp stability was observed.

## Figures and Tables

**Table 1:** UV thermal melting temperatures (*T*_m_^3^-values) of the triple helices.

Duplex	ON3	ON4	ON5	ON6	ON7	ON8
*Target 1* (pH 6.0)	32.3	36.8 (+4.5)	34.5 (+2.2)	35.2 (+2.9)	34.9 (+2.6)	36.1 (+3.8)
*Target 1* (pH 7.0)	20.2	24.2 (+4.0)	23.2 (+3.0)	25.3 (+5.1)	23.0 (+2.8)	24.5 (+4.3)
*Target 2* (pH 6.0)	31.1	36.9 (+5.8)	34.8 (+3.7)	34.3 (+3.2)	n.a.	n.a.
*Target 2* (pH 7.0)	19.4	25.2 (+5.8)	24.2 (+4.8)	24.9 (+5.5)	n.a.	n.a.

Notes: ∆*T*_m_^3^-values are shown in parentheses in comparison to the *T*_m_^3^-values of *Target 1 or Target 2* in the presence of **ON3**. *T*_m_^3^-values are average values extracted from three 0.2 °C min^−1^ heating and cooling ramps. Error limit in each measurement is in maximum ±0.3 °C.

## Supplementary Materials

The supplementary materials are available online.
