# Supplementary material for: Synthesis of Aminoglycoside-2′-O-Methyl Oligoribonucleotide Fusions
Source: Molecules. 2017 May 8;22(5):760. doi: 10.3390/molecules22050760 (PMC6154110; doi:10.3390/molecules22050760)
Supplement: Supplementary file 1 [file molecules-22-00760-s001.pdf]

Supporting Information to:

Synthesis of Aminoglycoside-2′-*O*-Methyl Oligoribonucleotide Fusions

Lotta Granqvist[1], Andrzej Kraszewski [2,3], Ville Tähtinen[1] and Pasi Virta[1,*]

[1] Department of Chemistry, University of Turku, Vatselankatu 2, 20014 Turku, Finland
[2] Centre of New Technologies, University of Warsaw, Banacha 2c, 02 097 Warsaw; Poland
[3] College of Inter-Faculty Individual Studies in Mathematics and Natural Sciences, University of Warsaw, Banacha 2c, 02 097 Warsaw; Poland
* Correspondence: pamavi@utu.fi; Tel.: +358 294 503 209

Contents

Figure S1. ^{1}H NMR (500 MHz, CD_3OD) spectrum of **11**. S3
Figure S2. ^{1}H NMR (500 MHz, CD_3OD) spectrum of **11**. S3
Figure S3. ^{13}C NMR (125 MHz, CD_3OD) spectrum of **11**. S4
Figure S4. ^{1}H NMR (500 MHz, CD_3OD) spectrum of **12**. S4
Figure S5. ^{1}H NMR (500 MHz, CD_3OD) spectrum of **12**. S5
Figure S6. ^{13}C NMR (125 MHz, CD_3OD) spectrum of **12**. S5
Figure S7. ^{1}H NMR (500 MHz, CD_3OD) spectrum of **13**. S6
Figure S8. ^{1}H NMR (500 MHz, CD_3OD) spectrum of **13**. S6
Figure S9. ^{13}C NMR (125 MHz, CD_3OD) spectrum of **13**. S7
Figure S10. ^{1}H NMR (500 MHz, CD_3OD) spectrum of **14**. S7
Figure S11. ^{1}H NMR (500 MHz, CD_3OD) spectrum of **14**. S8
Figure S12. ^{13}C NMR (125 MHz, CD_3OD) spectrum of **14**. S8
Figure S13. ^{1}H NMR (500 MHz, CD_3OD) spectrum of **15**. S9
Figure S14. ^{1}H NMR (500 MHz, CD_3OD) spectrum of **15**. S9
Figure S15. ^{13}C NMR (125 MHz, CD_3OD) spectrum of **15**. S10
Figure S16. ^{13}C NMR (125 MHz, CD_3OD) spectrum of **15**. S10
Figure S17. ^{1}H NMR (500 MHz, CD_3OD) spectrum of **16**. S11
Figure S18. ^{1}H NMR (500 MHz, CD_3OD) spectrum of **16**. S11
Figure S19. ^{13}C NMR (125 MHz, CD_3OD) spectrum of **16**. S12
Figure S20. ^{13}C NMR (125 MHz, CD_3OD) spectrum of **16**. S12
Figure S21. ^{1}H NMR (500 MHz, CD_3OD) spectrum of **17**. S13
Figure S22. ^{1}H NMR (500 MHz, CD_3OD) spectrum of **17**. S13
Figure S23. ^{13}C NMR (125 MHz, CD_3OD) spectrum of **17**. S14
Figure S24. ^{1}H NMR (500 MHz, CD_3OD) spectrum of **18**. S14
Figure S25. ^{1}H NMR (500 MHz, CD_3OD) spectrum of **18**. S15
Figure S26. ^{13}C NMR (125 MHz, CD_3OD) spectrum of **18**. S15
Figure S27. ^{1}H NMR (500 MHz, CD_3OD) spectrum of **19**. S16
Figure S28. ^{1}H NMR (500 MHz, CD_3OD) spectrum of **19**. S16
Figure S29. ^{1}H NMR (500 MHz, CD_3OD) spectrum of **19**. S17
Figure S30. ^{1}H NMR (500 MHz, CD_3OD) spectrum of **19**. S17
Figure S31. ^{13}C NMR (125 MHz, CD_3OD) spectrum of **19**. S18
Figure S32. HSQC spectrum of **19**. S18
Figure S33. ^{1}H NMR (500 MHz, CD_3OD) spectrum of **20**. S19
Figure S34. ^{1}H NMR (500 MHz, CD_3OD) spectrum of **20**. S19
Figure S35. ^{1}H NMR (500 MHz, CD_3OD) spectrum of **20**. S20

Figure S36. ^{1}H NMR (500 MHz, CD_3OD) spectrum of **20**. S20
Figure S37. ^{13}C NMR (125 MHz, CD_3OD) spectrum of **20**. S21
Figure S38. HSQC spectrum of **20**. S21
Figure S39. ^{31}P NMR (200 MHz, CD_3CN) spectrum of **3**. S22
Figure S40. ^{1}H NMR (500 MHz, CD_3CN) spectrum of **3**. S22
Figure S41. ^{1}H NMR (500 MHz, CD_3CN) spectrum of **3**. S23
Figure S42. ^{1}H NMR (500 MHz, CD_3CN) spectrum of **3**. S23
Figure S43. ^{13}C NMR (125 MHz, CD_3CN) spectrum of **3**. S24
Figure S44. ^{31}P NMR (200 MHz, CD_3CN) spectrum of **4.** S24
Figure S45. ^{1}H NMR (500 MHz, CD_3CN) spectrum of **4.** S25
Figure S46. ^{1}H NMR (500 MHz, CD_3CN) spectrum of **4.** S25
Figure S47. ^{1}H NMR (500 MHz, CD_3CN) spectrum of **4.** S26
Figure S48. ^{13}C NMR (125 MHz, CD_3CN) spectrum of **4.** S26
Table S1. (MS ESI-TOF) data of the oligonucleotides S27

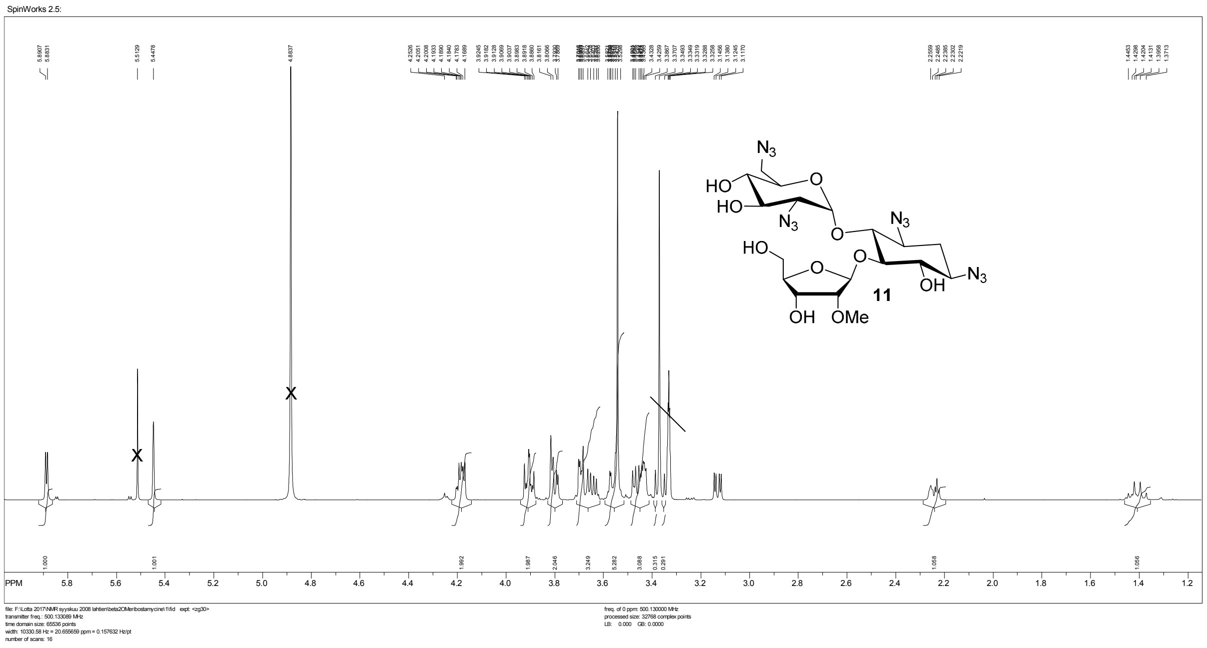

Figure S1. ^{1}H NMR (500 MHz, CD_3OD) spectrum of **11**.

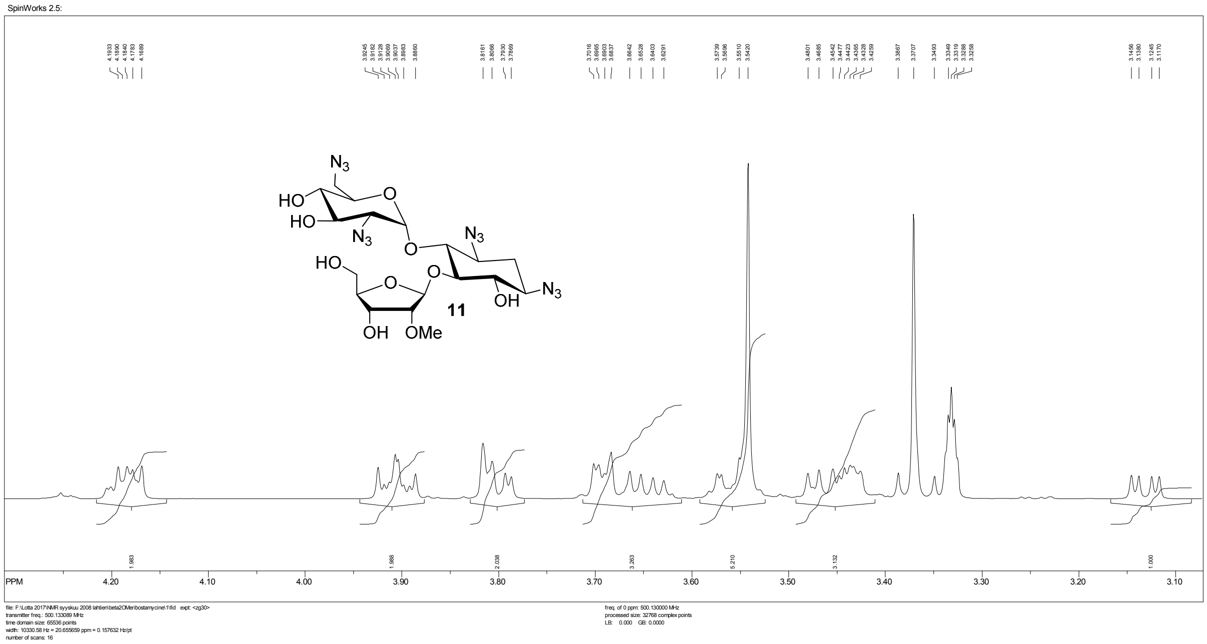

Figure S2. ^{1}H NMR (500 MHz, CD_3OD) spectrum of **11**.

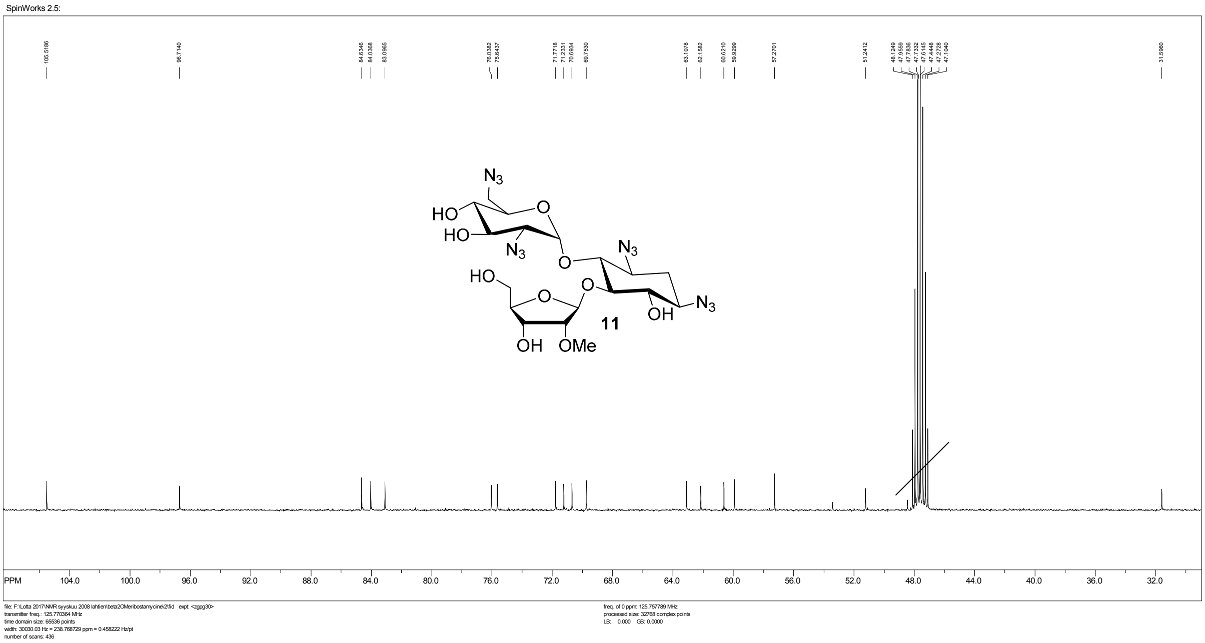

Figure S3. ^{13}C NMR (125 MHz, CD_3OD) spectrum of **11**.

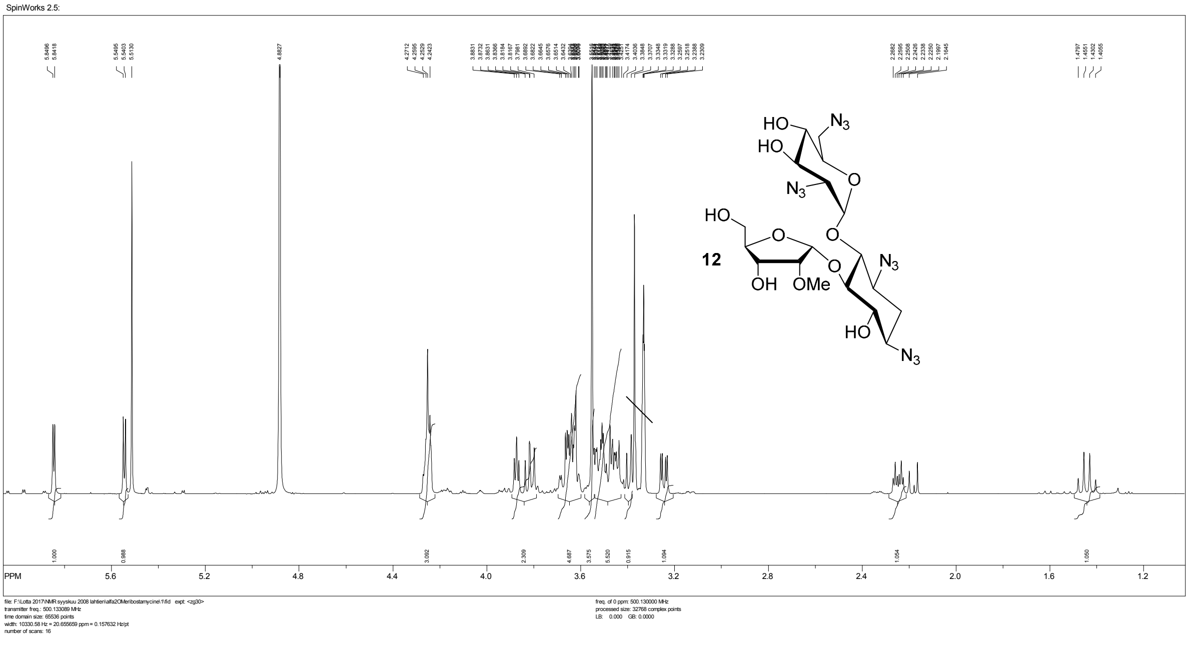

Figure S4. ^{1}H NMR (500 MHz, CD_3OD) spectrum of **12**.

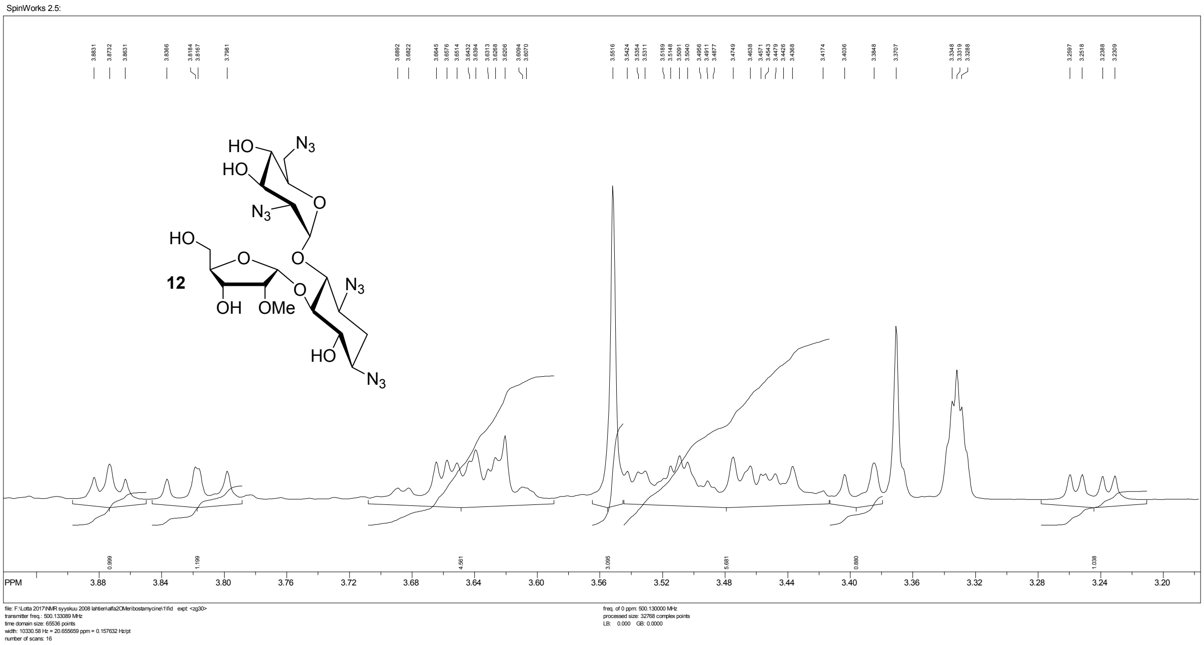

Figure S5. ^{1}H NMR (500 MHz, CD_3OD) spectrum of **12**.

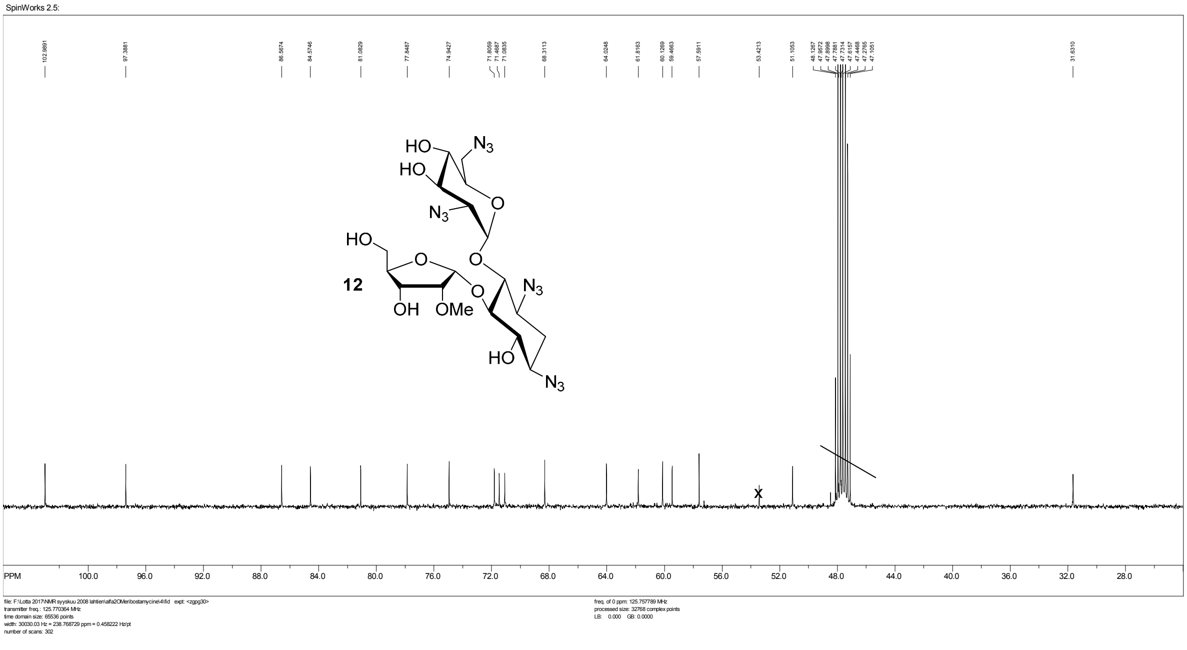

Figure S6. ^{13}C NMR (125 MHz, CD_3OD) spectrum of **12**.

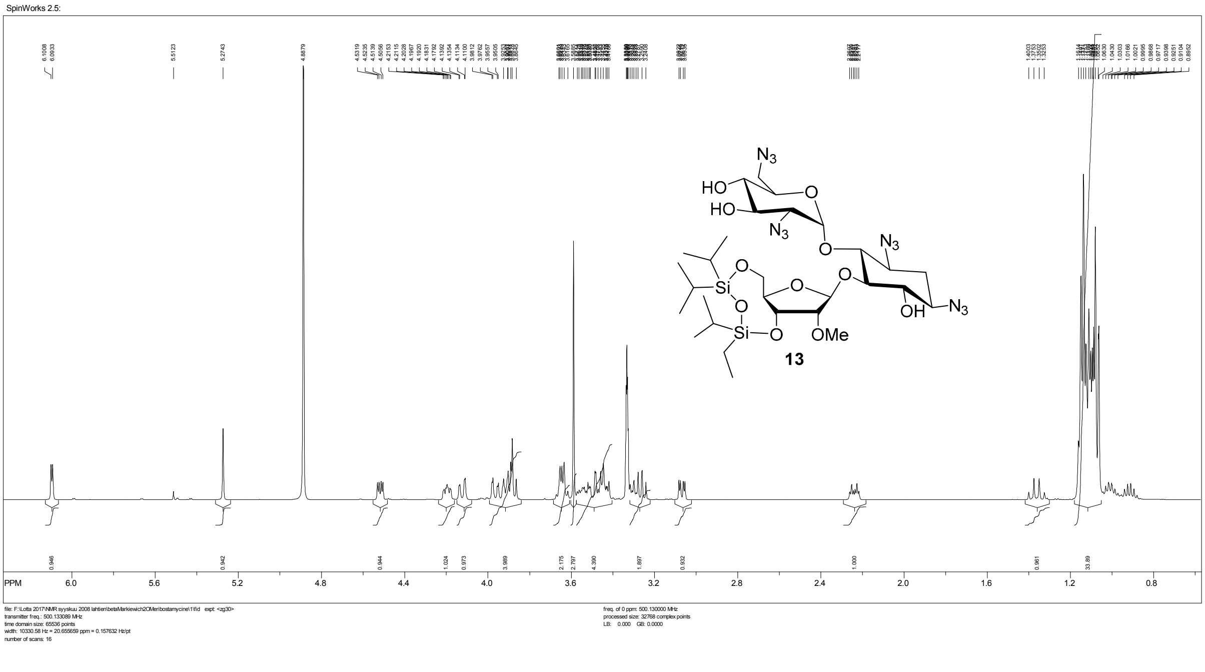

Figure S7. ^{1}H NMR (500 MHz, CD_3OD) spectrum of **13**.

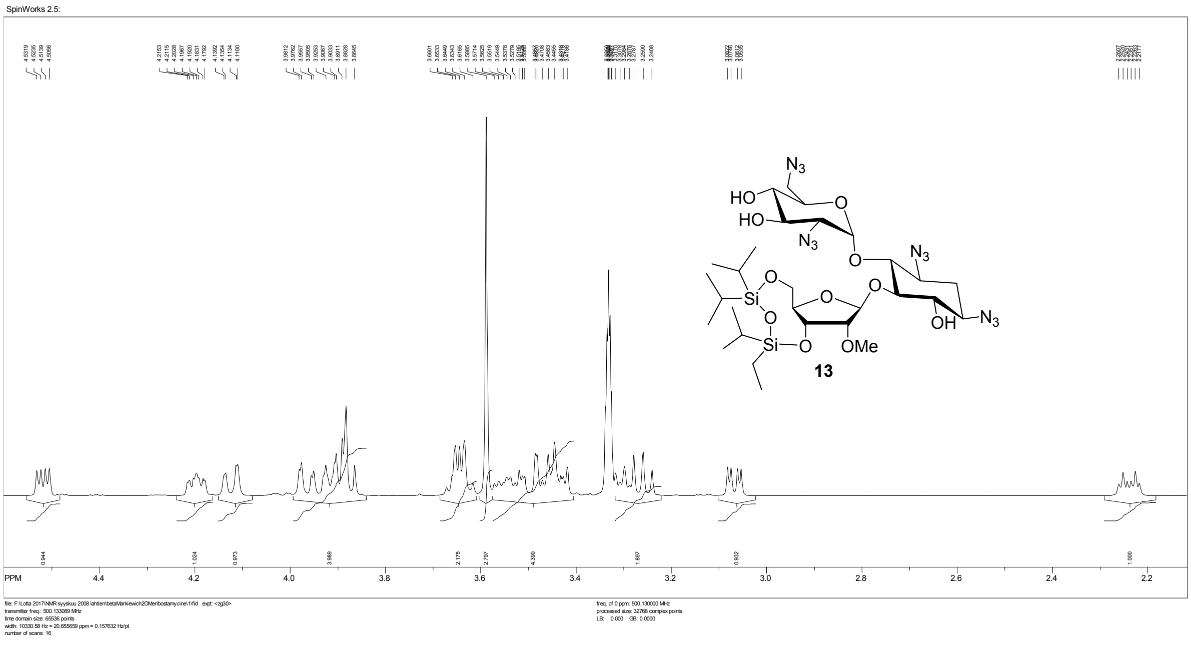

Figure S8. ^{1}H NMR (500 MHz, CD_3OD) spectrum of **13**.

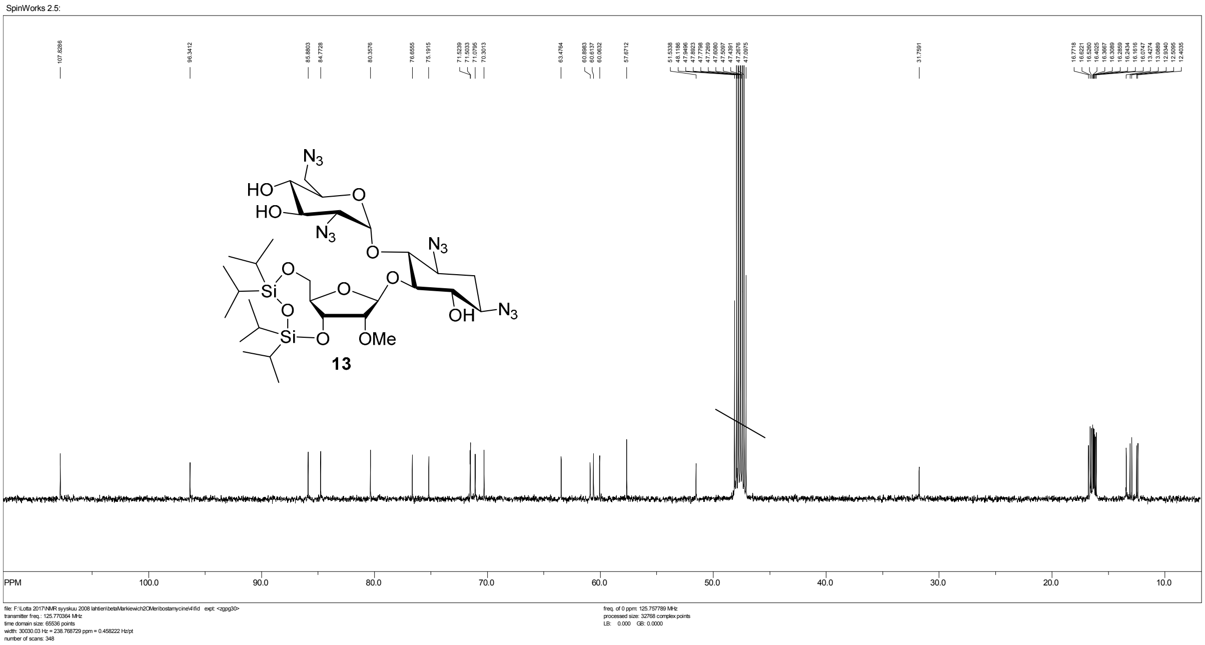

Figure S9. ^{13}C NMR (125 MHz, CD_3OD) spectrum of **13**.

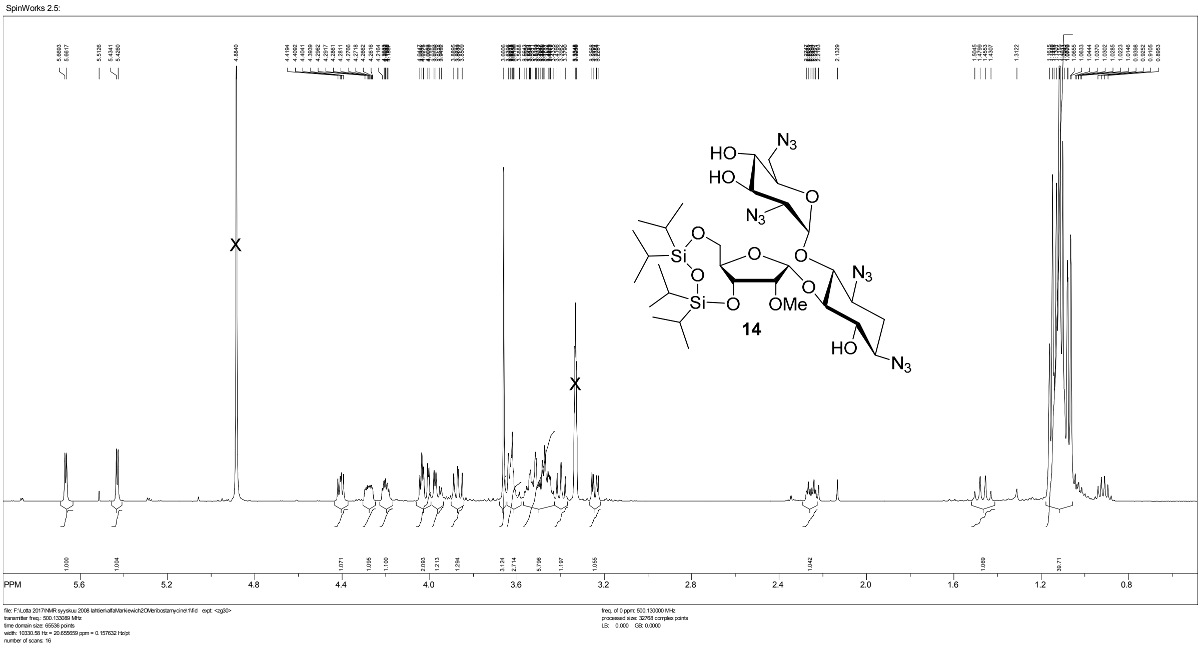

Figure S10. ^{1}H NMR (500 MHz, CD_3OD) spectrum of **14**.

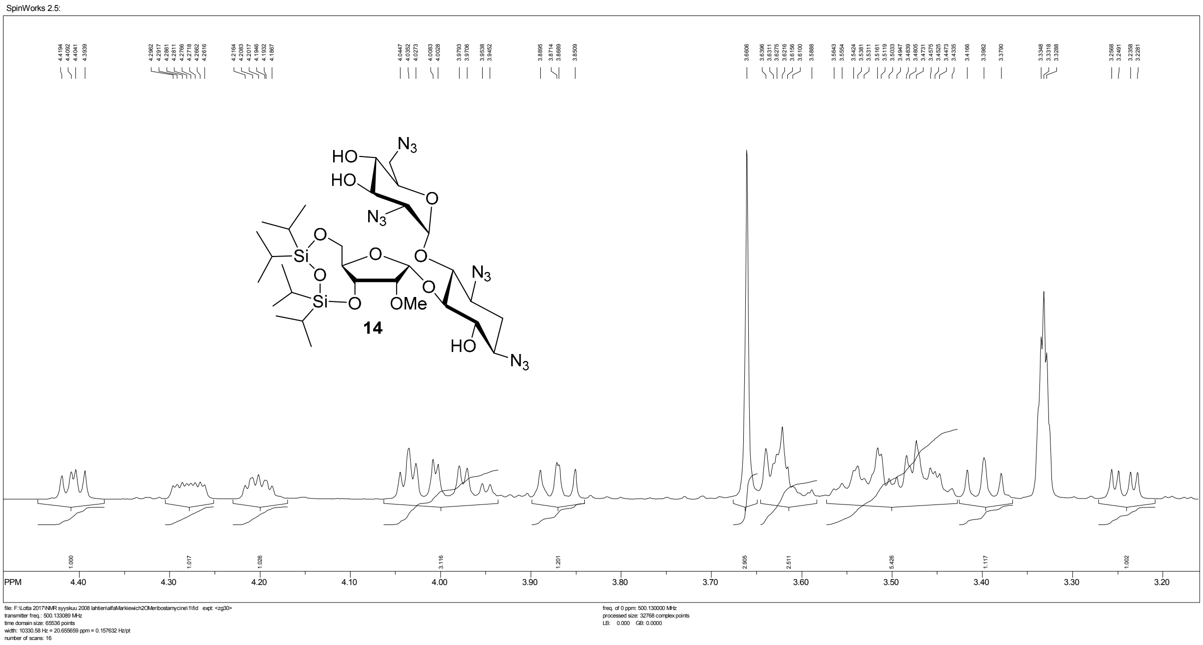

Figure S11. ^{1}H NMR (500 MHz, CD_3OD) spectrum of **14**.

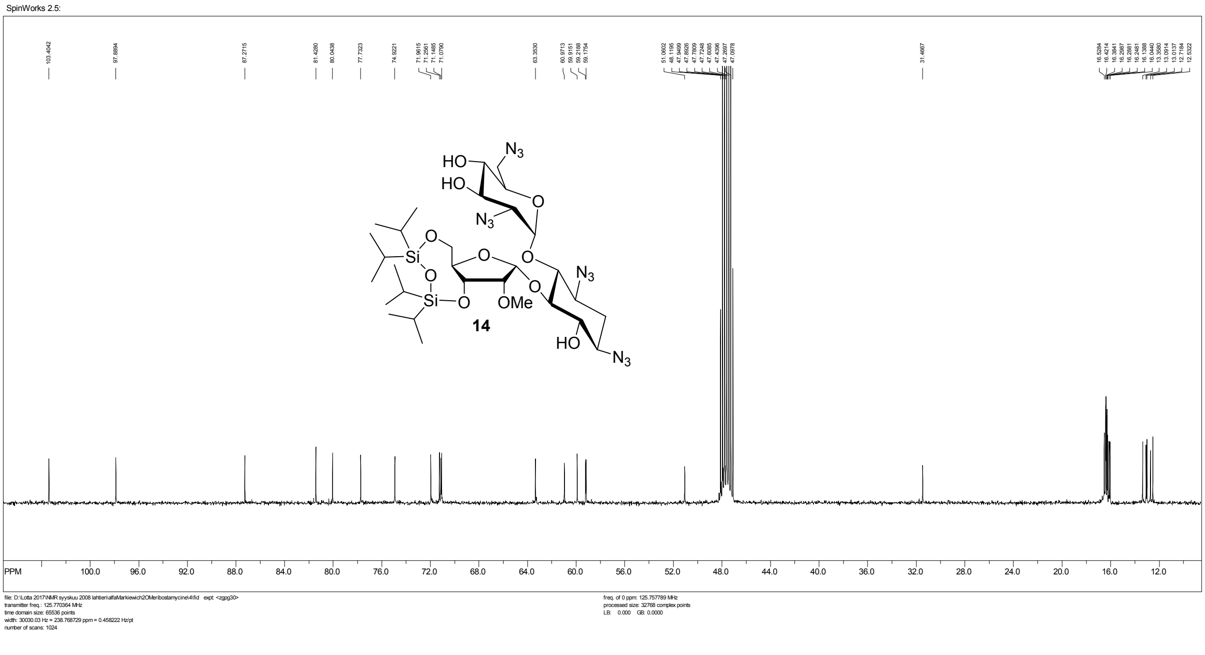

Figure S12. ^{13}C NMR (125 MHz, CD_3OD) spectrum of **14**.

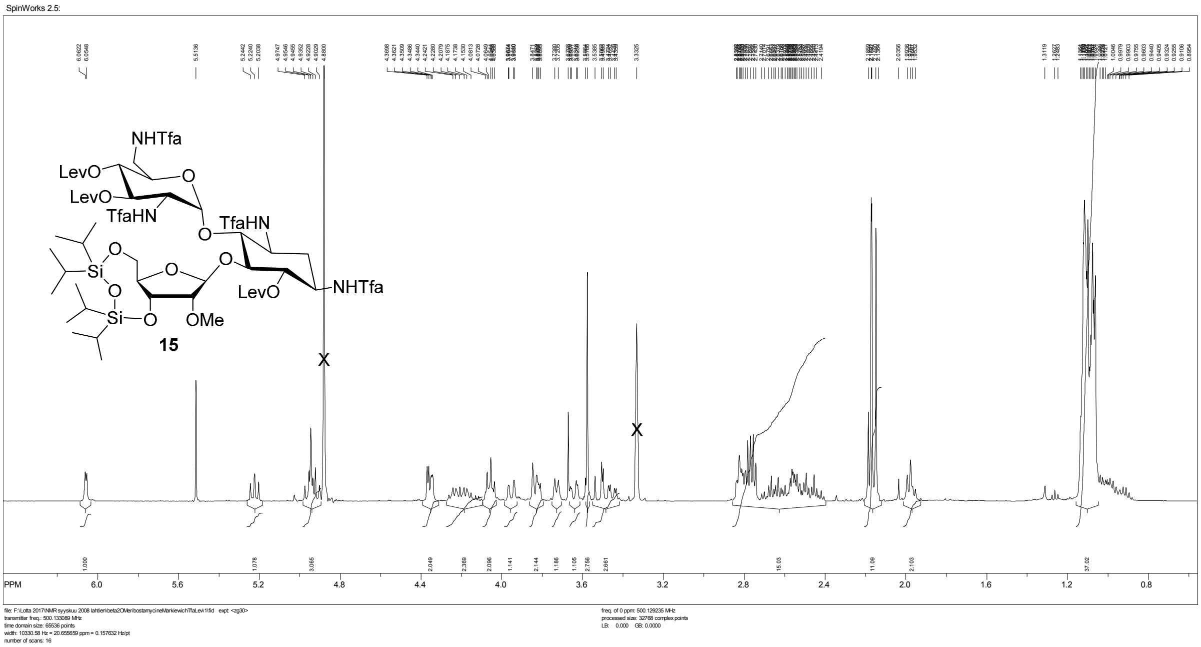

Figure S13. ^{1}H NMR (500 MHz, CD_3OD) spectrum of **15**.

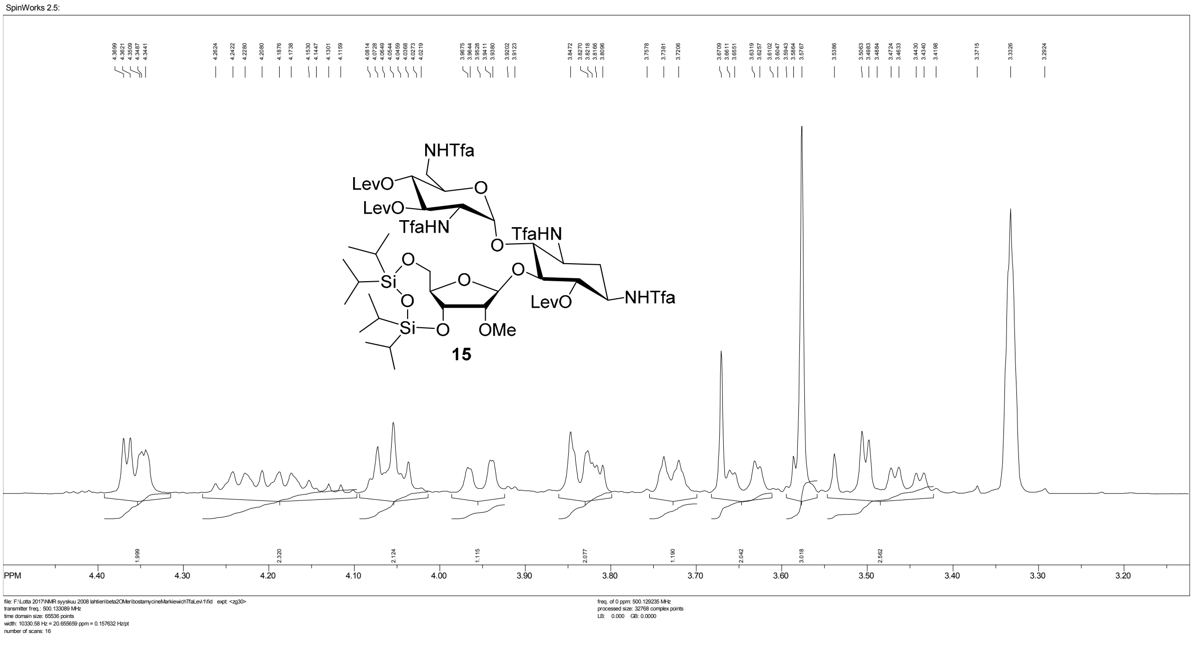

Figure S14. ^{1}H NMR (500 MHz, CD_3OD) spectrum of **15**.

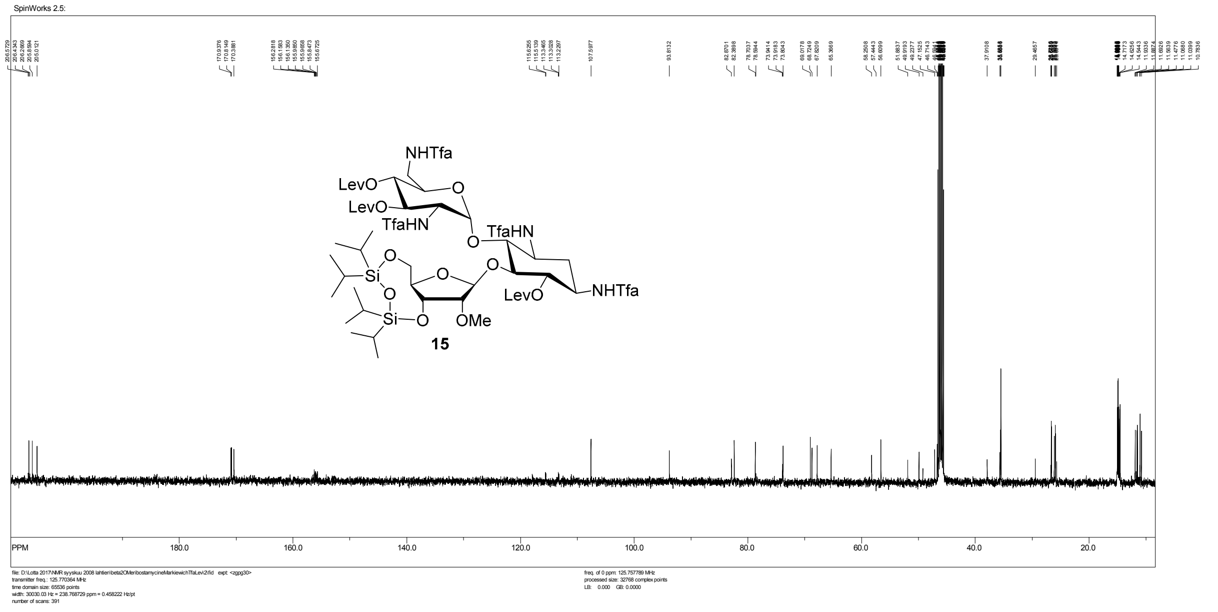

Figure S15. ^{13}C NMR (125 MHz, CD_3OD) spectrum of **15**.

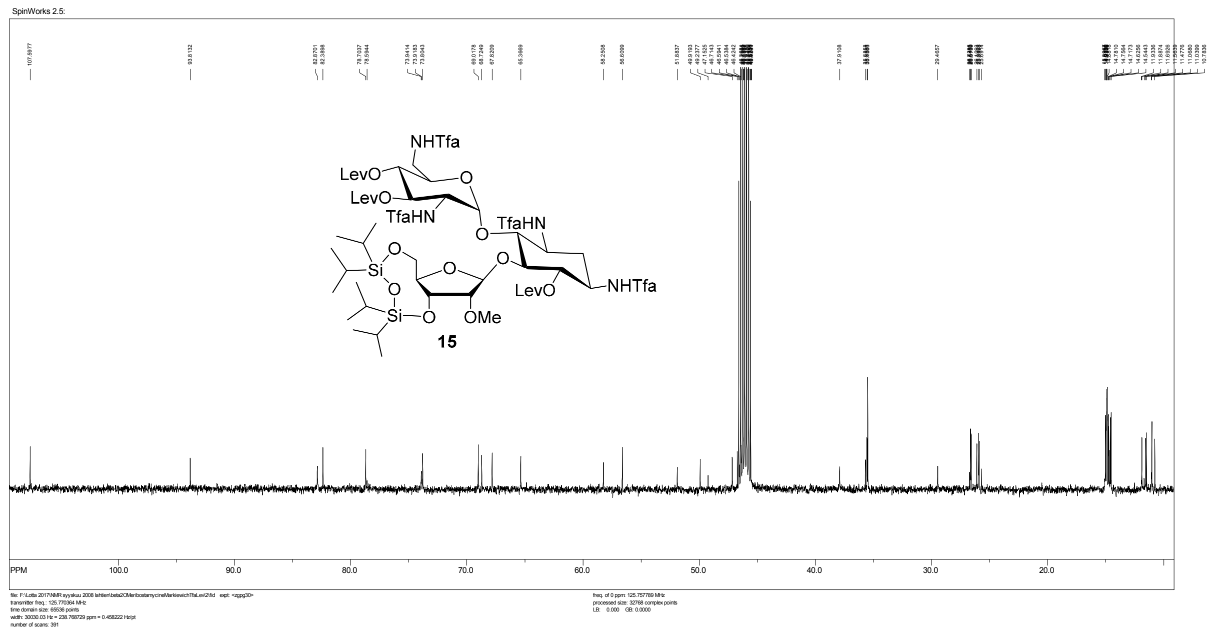

Figure S16. ^{13}C NMR (125 MHz, CD_3OD) spectrum of **15**.

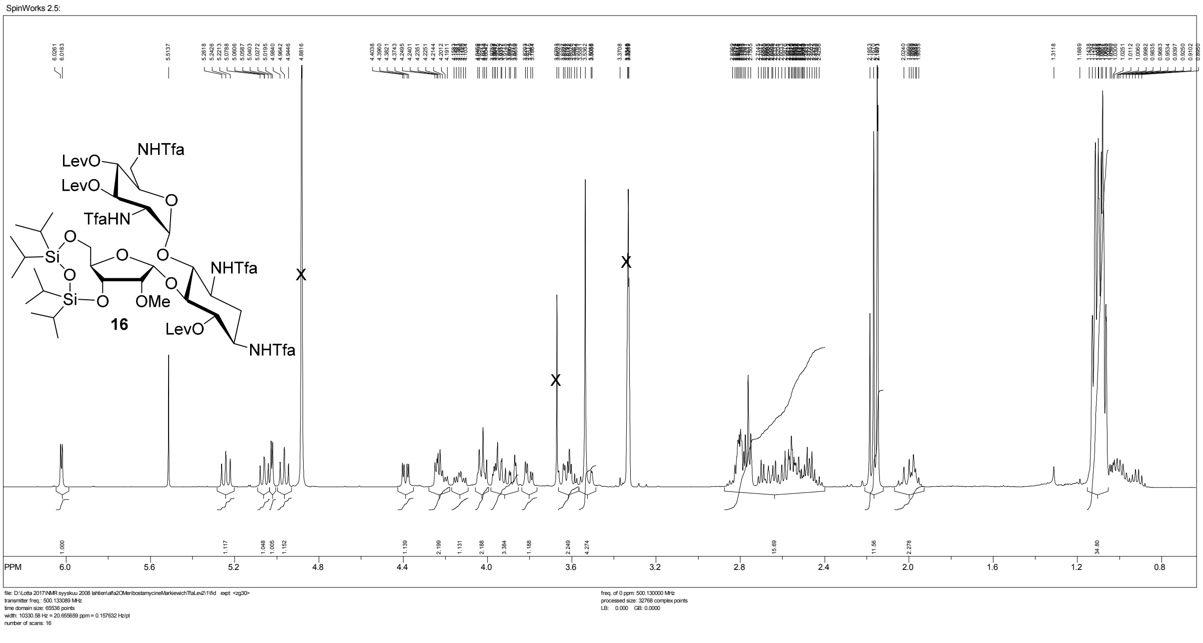

Figure S17. ^{1}H NMR (500 MHz, CD_3OD) spectrum of **16**.

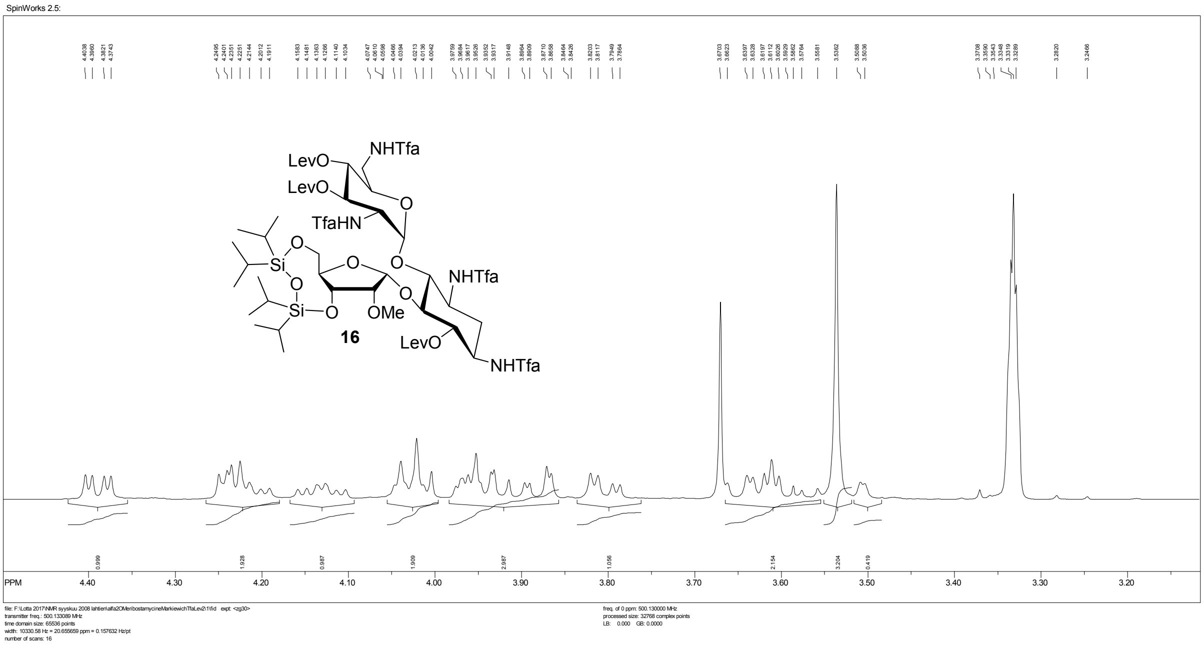

Figure S18. ^{1}H NMR (500 MHz, CD_3OD) spectrum of **16**.

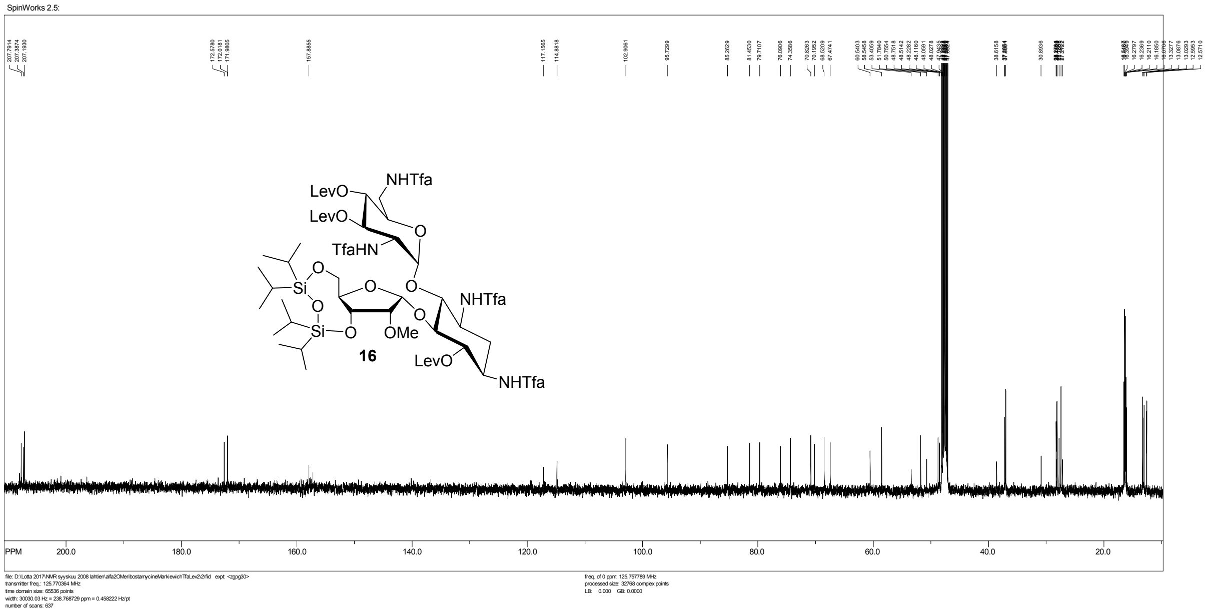

Figure S19. ^{13}C NMR (125 MHz, CD_3OD) spectrum of **16**.

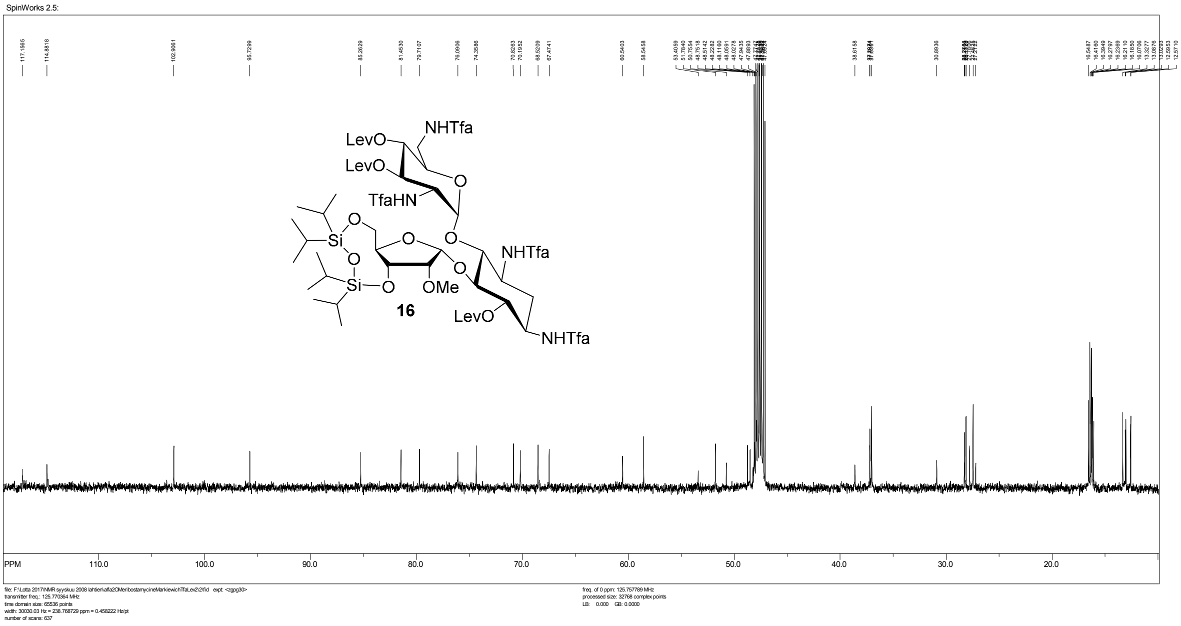

Figure S20. ^{13}C NMR (125 MHz, CD_3OD) spectrum of **16**.

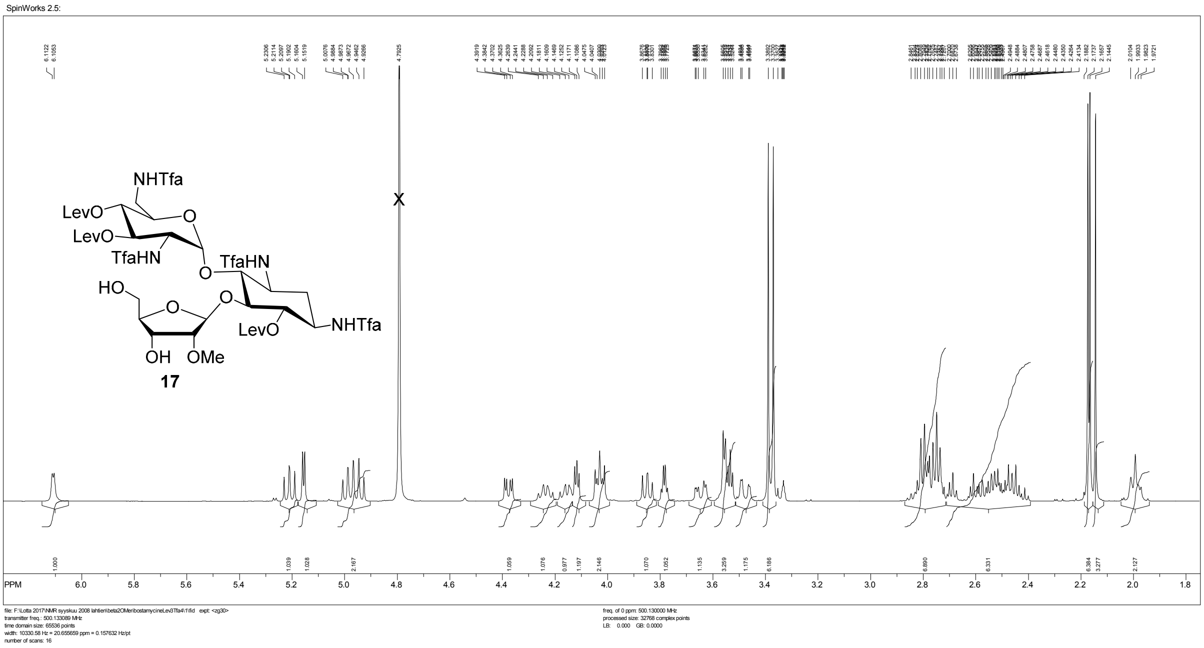

Figure S21. ^{1}H NMR (500 MHz, CD_3OD) spectrum of **17**.

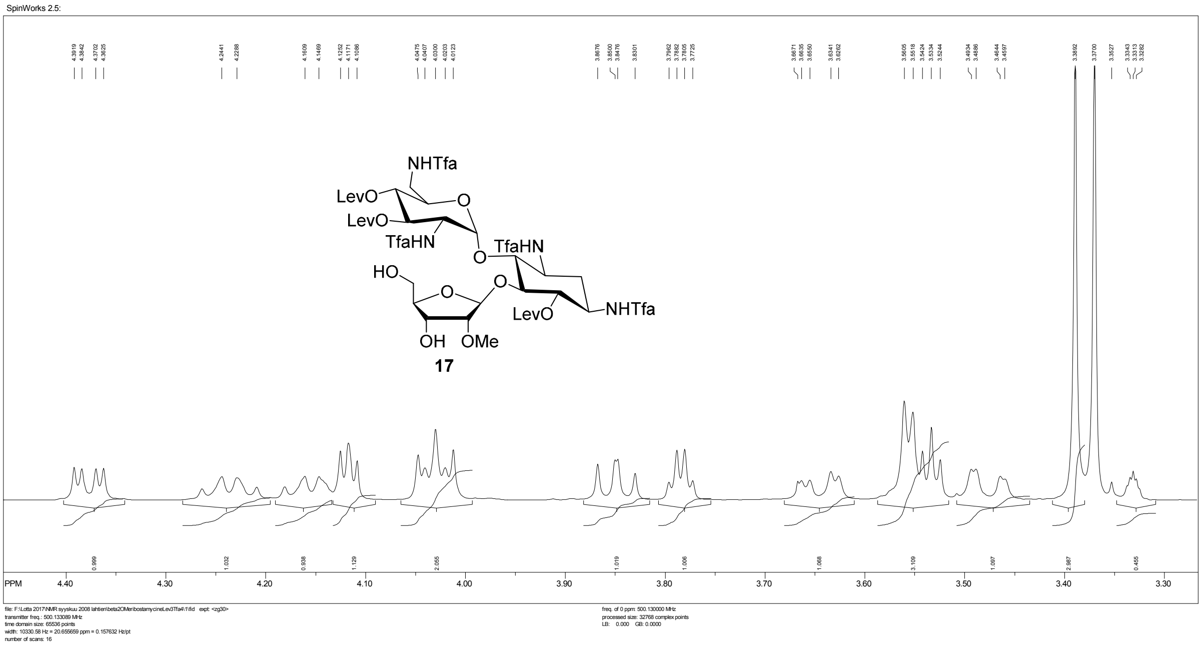

Figure S22. ^{1}H NMR (500 MHz, CD_3OD) spectrum of **17**.

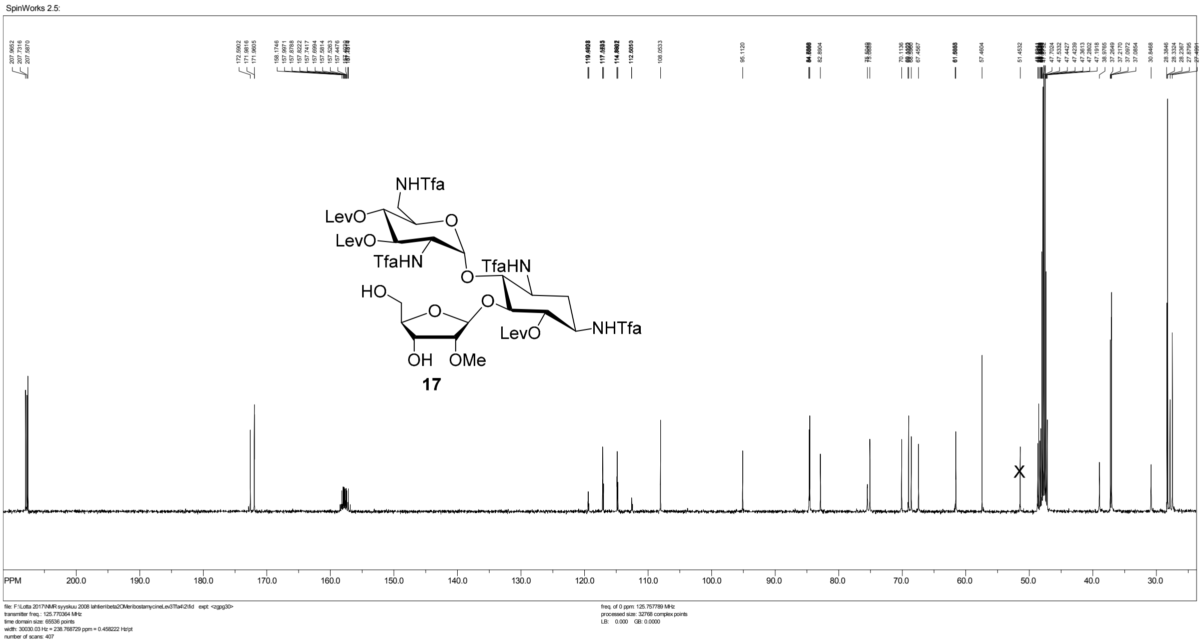

Figure S23. ^{13}C NMR (125 MHz, CD_3OD) spectrum of **17**.

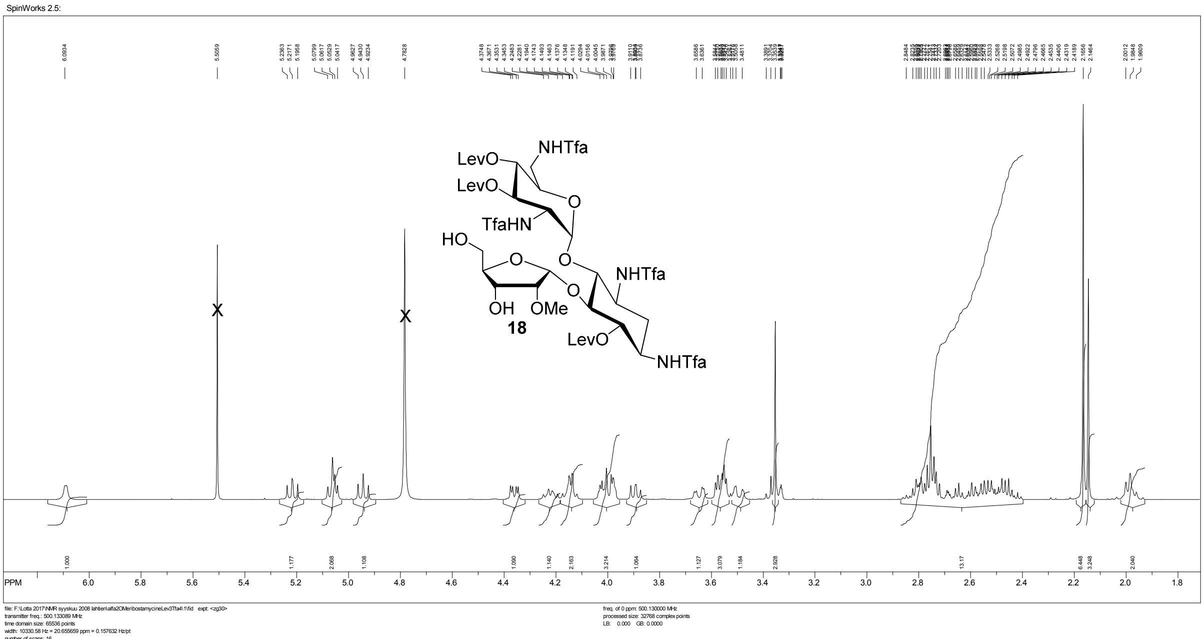

Figure S24. ^{1}H NMR (500 MHz, CD_3OD) spectrum of **18**.

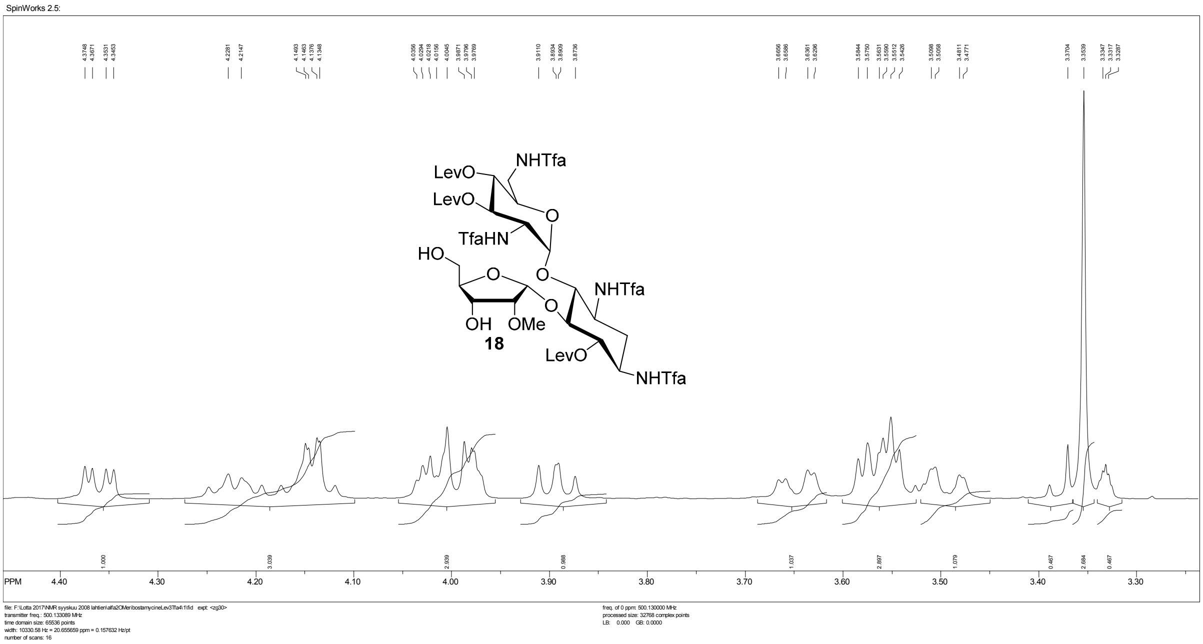

Figure S25. ^{1}H NMR (500 MHz, CD_3OD) spectrum of **18**.

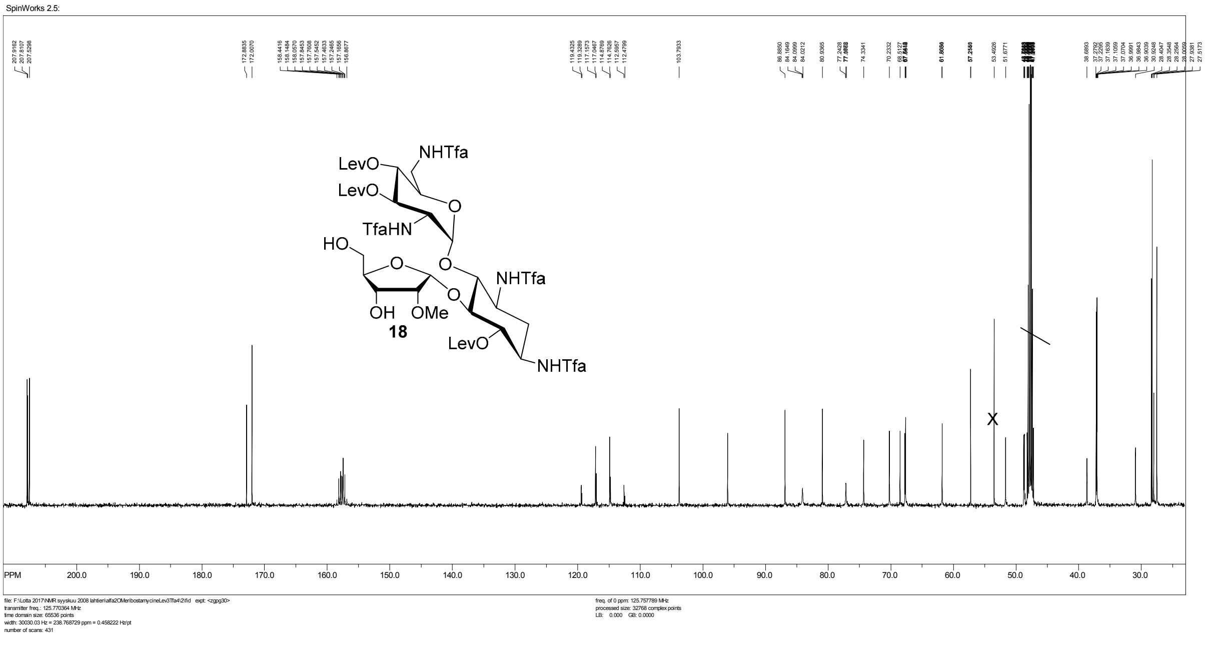

Figure S26. ^{13}C NMR (125 MHz, CD_3OD) spectrum of **18**.

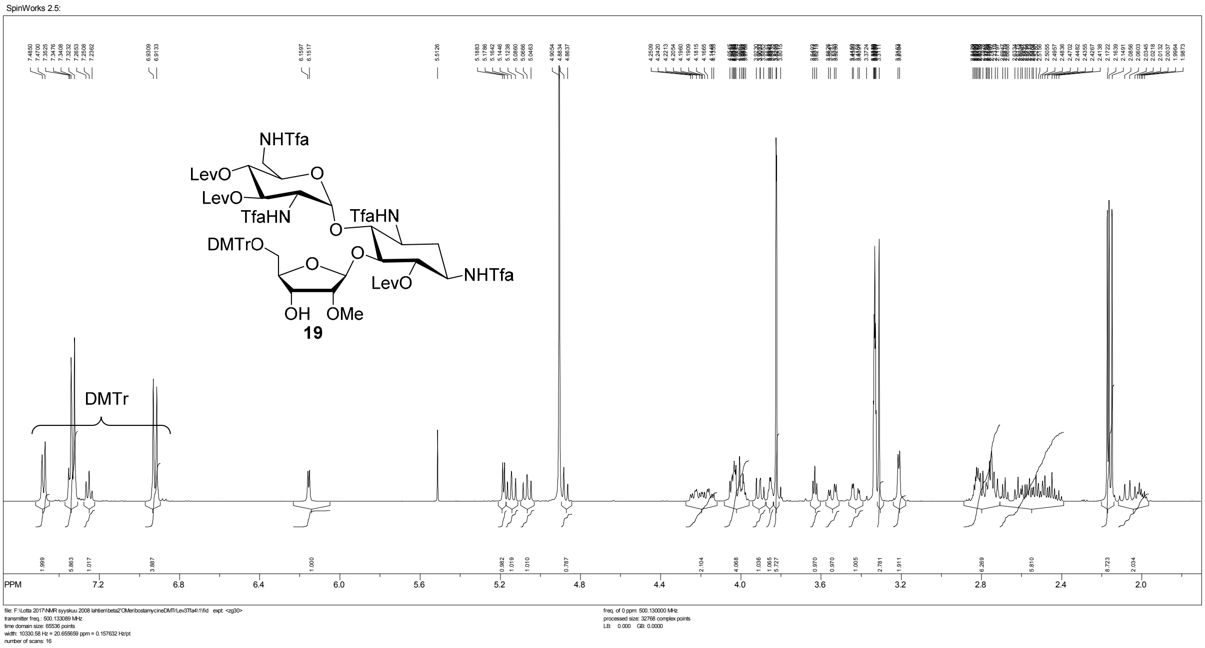

Figure S27. ^{1}H NMR (500 MHz, CD_3OD) spectrum of **19**.

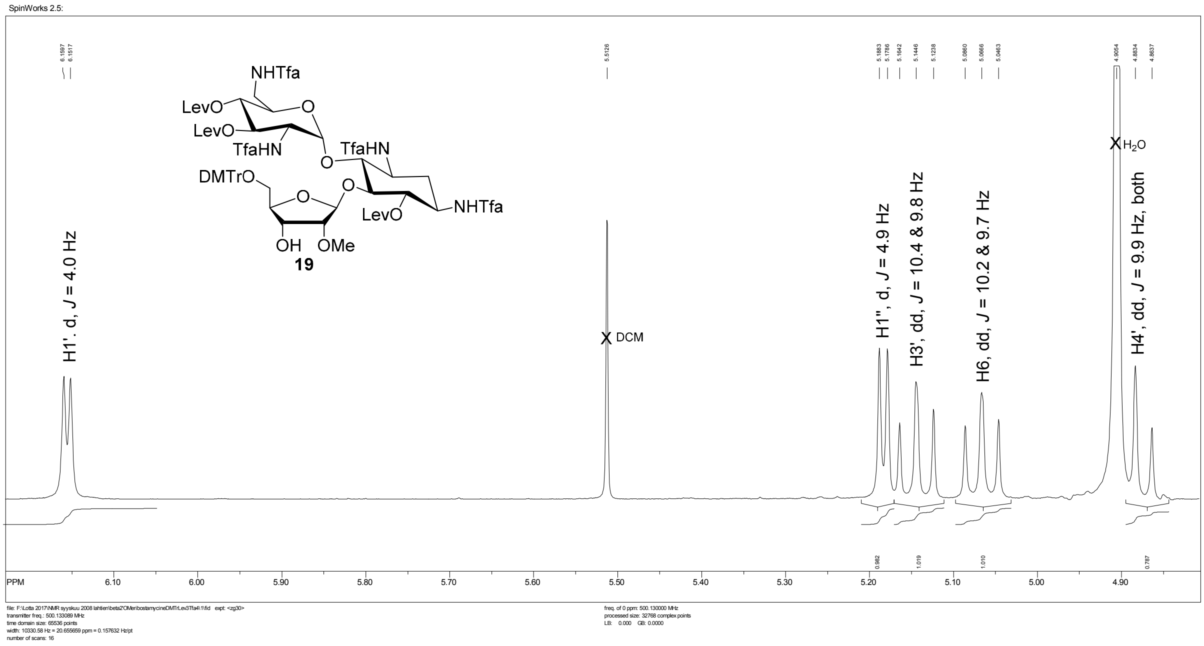

Figure S28. ^{1}H NMR (500 MHz, CD_3OD) spectrum of **19**.

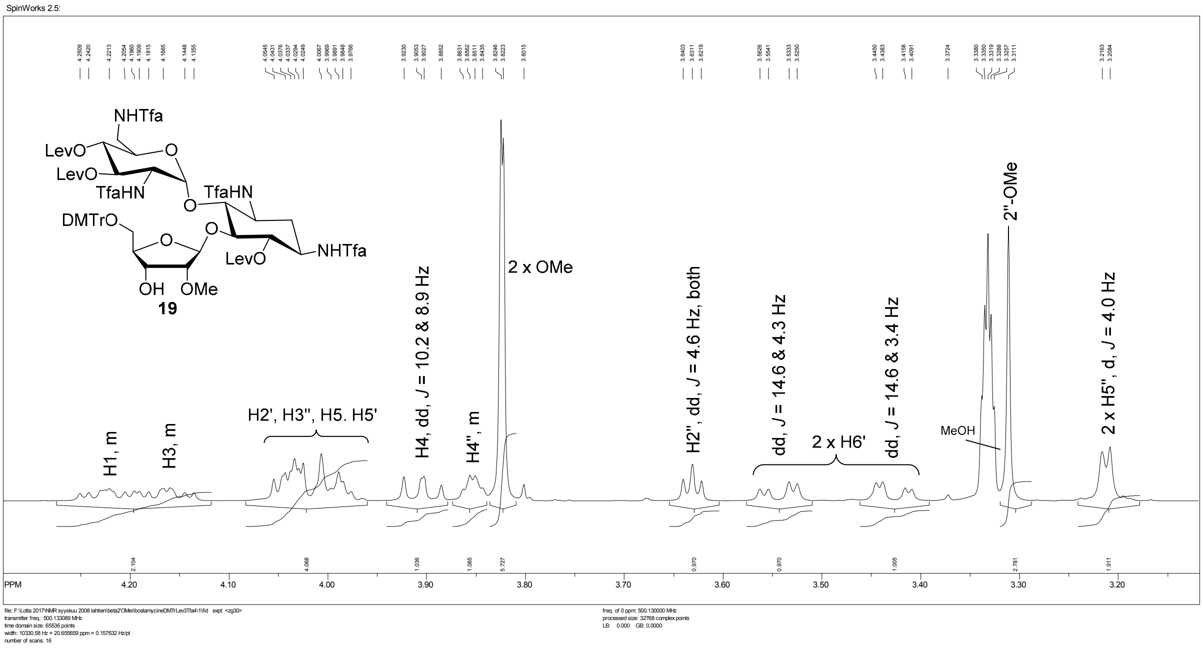

Figure S29. ^{1}H NMR (500 MHz, CD_3OD) spectrum of **19**.

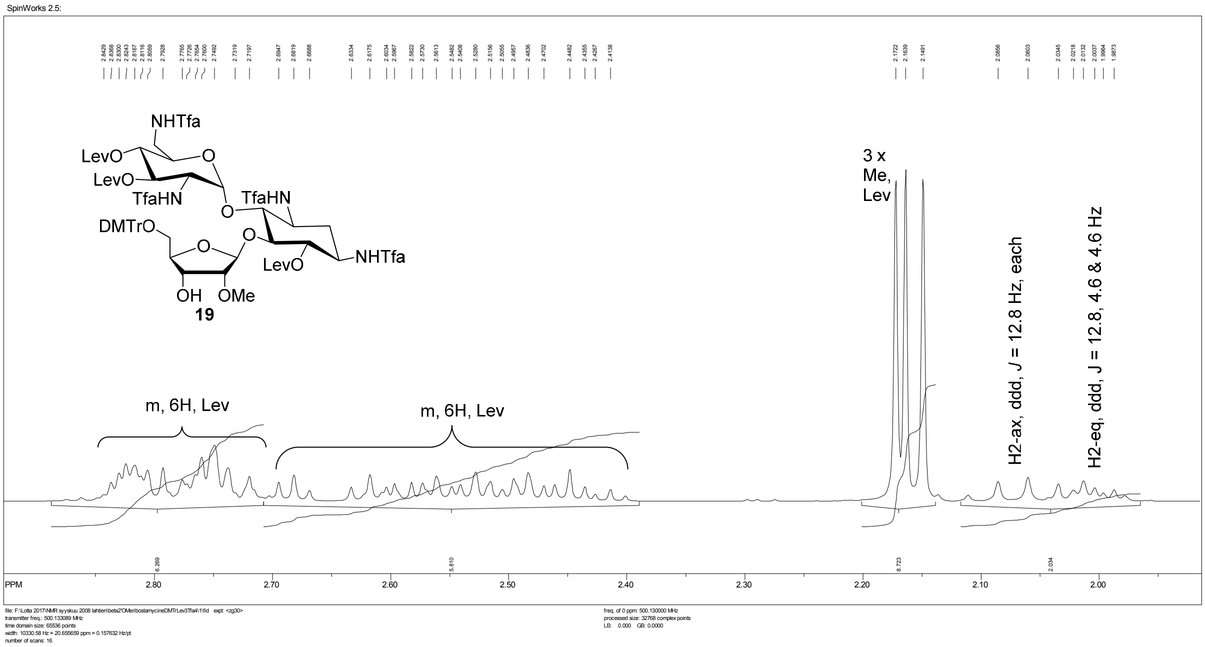

Figure S30. ^{1}H NMR (500 MHz, CD_3OD) spectrum of **19**.

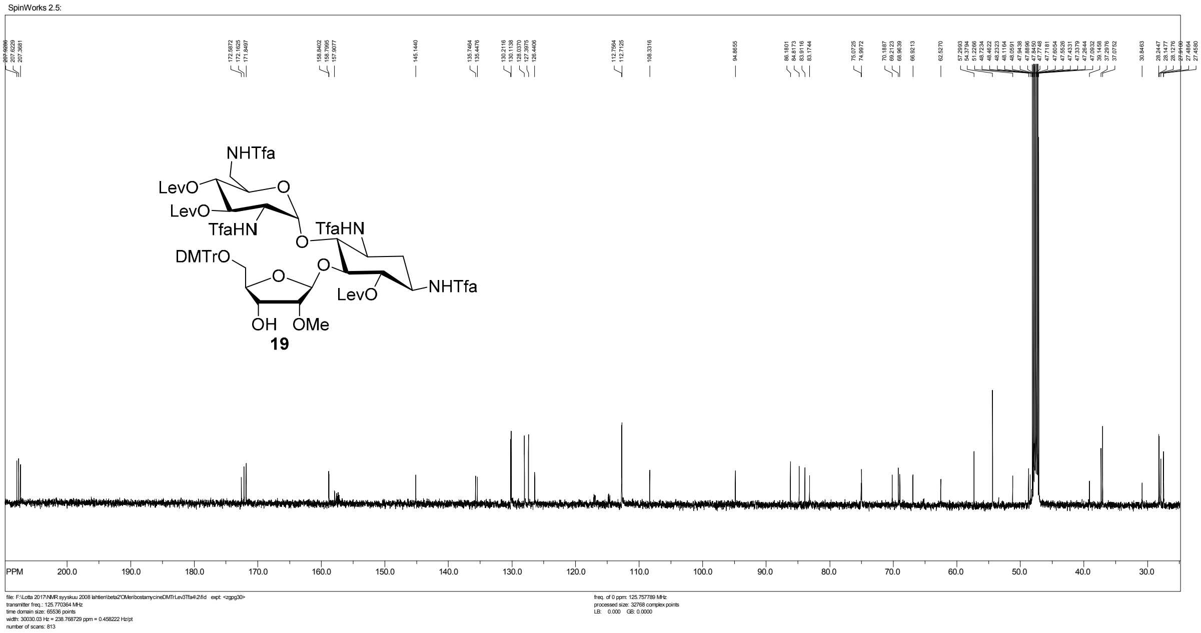

Figure S31. ^{13}C NMR (125 MHz, CD_3OD) spectrum of **19**.

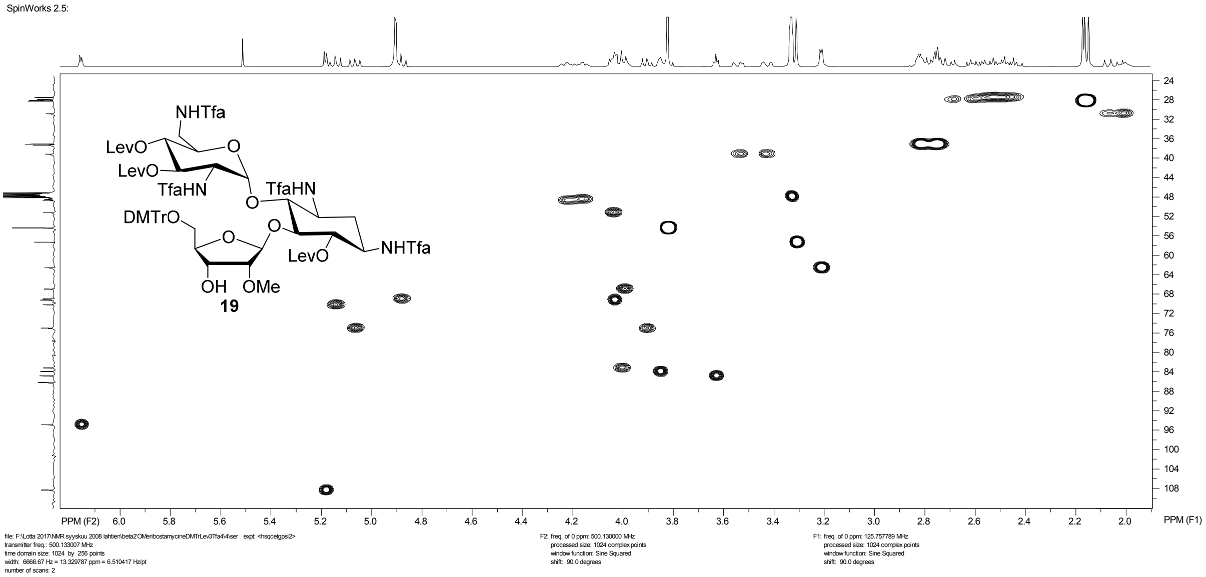

Figure S32. HSQC spectrum of **19**.

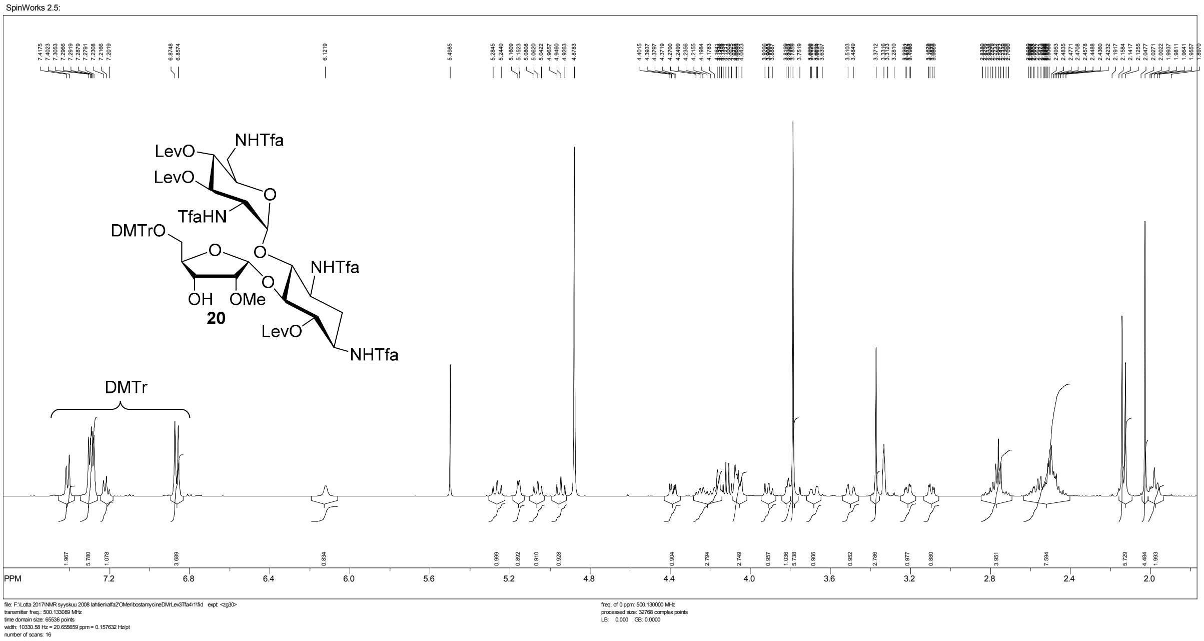

Figure S33. ^{1}H NMR (500 MHz, CD_3OD) spectrum of **20**.

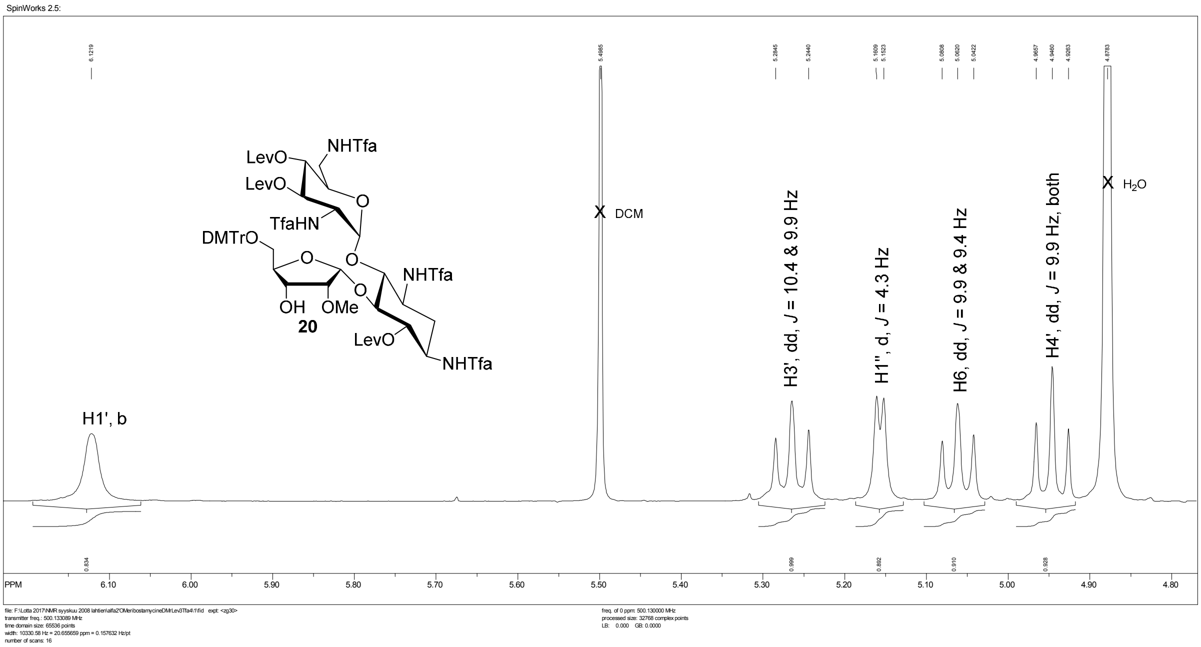

Figure S34. ^{1}H NMR (500 MHz, CD_3OD) spectrum of **20**.

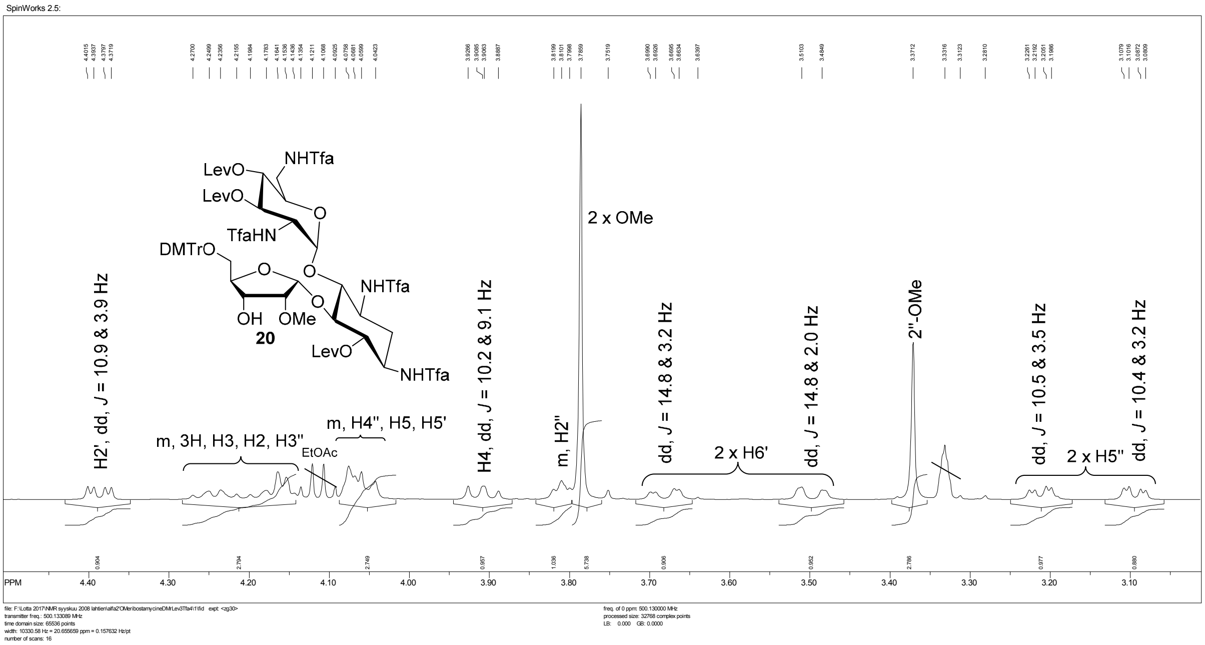

Figure S35. ^{1}H NMR (500 MHz, CD_3OD) spectrum of **20**.

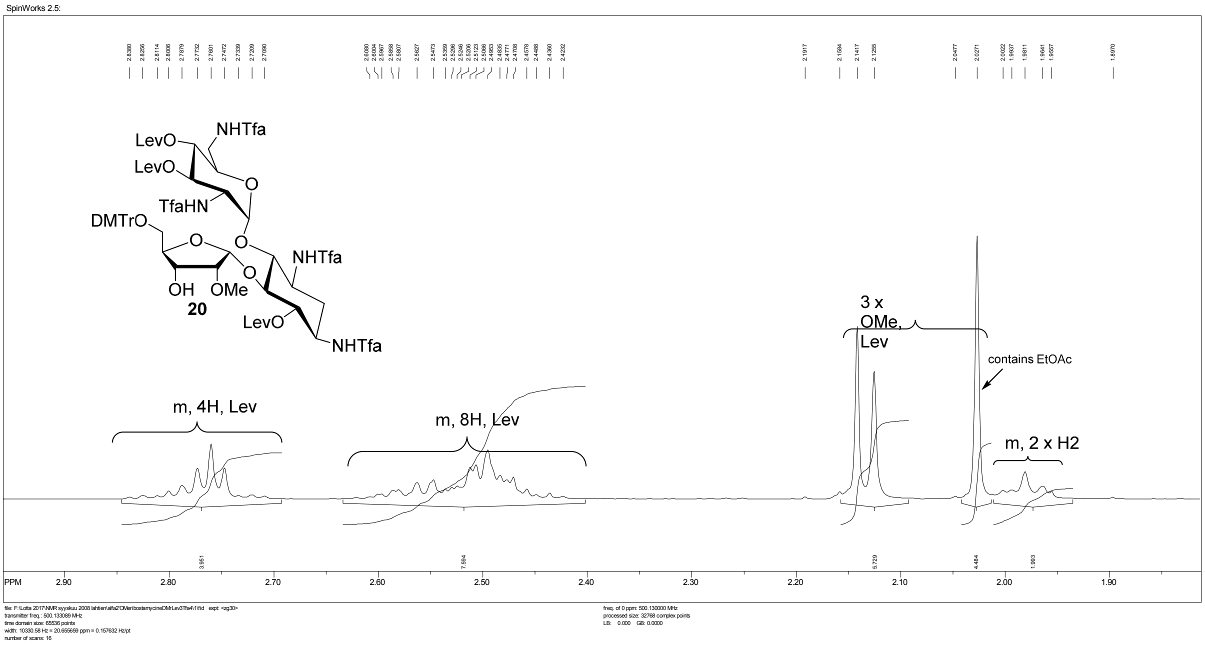

Figure S36. ^{1}H NMR (500 MHz, CD_3OD) spectrum of **20**.

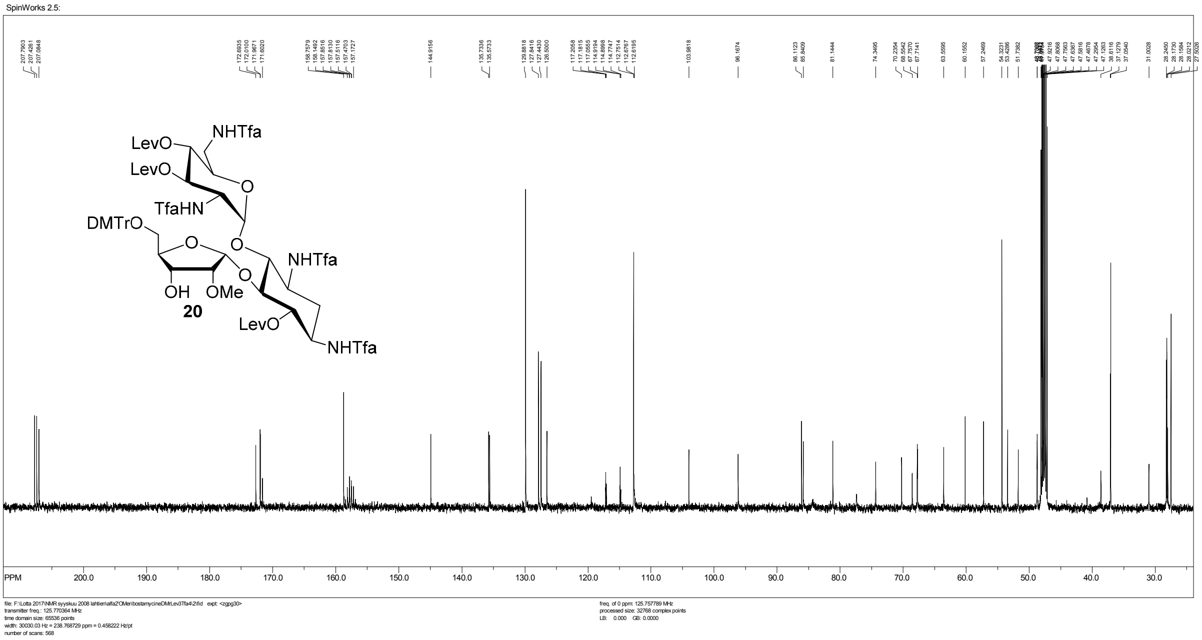

Figure S37. ^{13}C NMR (125 MHz, CD_3OD) spectrum of **20**.

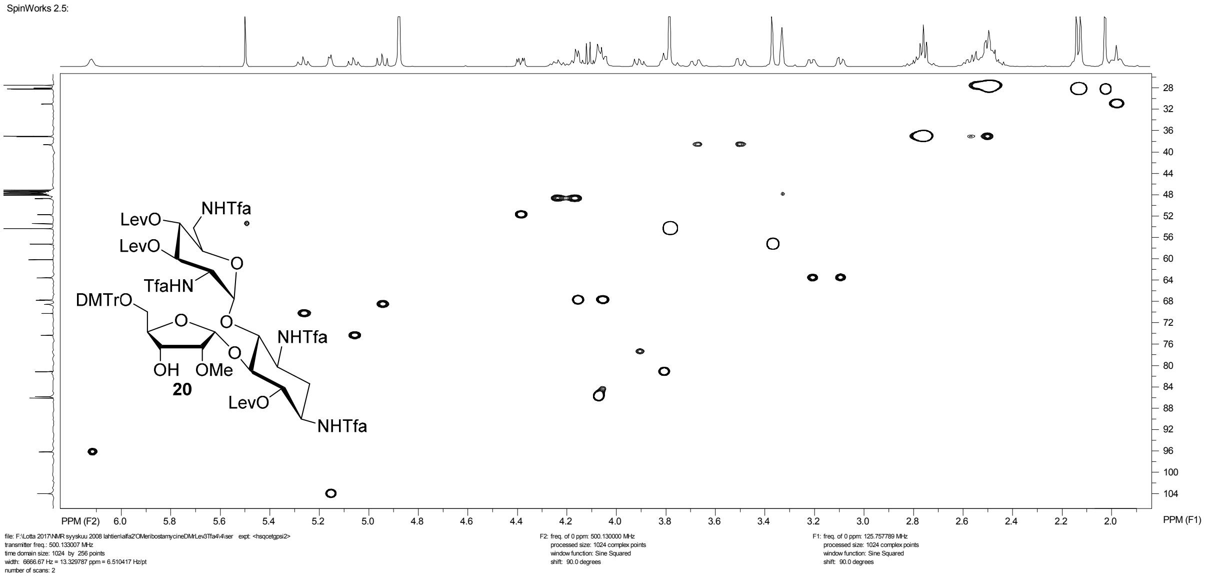

Figure S38. HSQC spectrum of **20**.

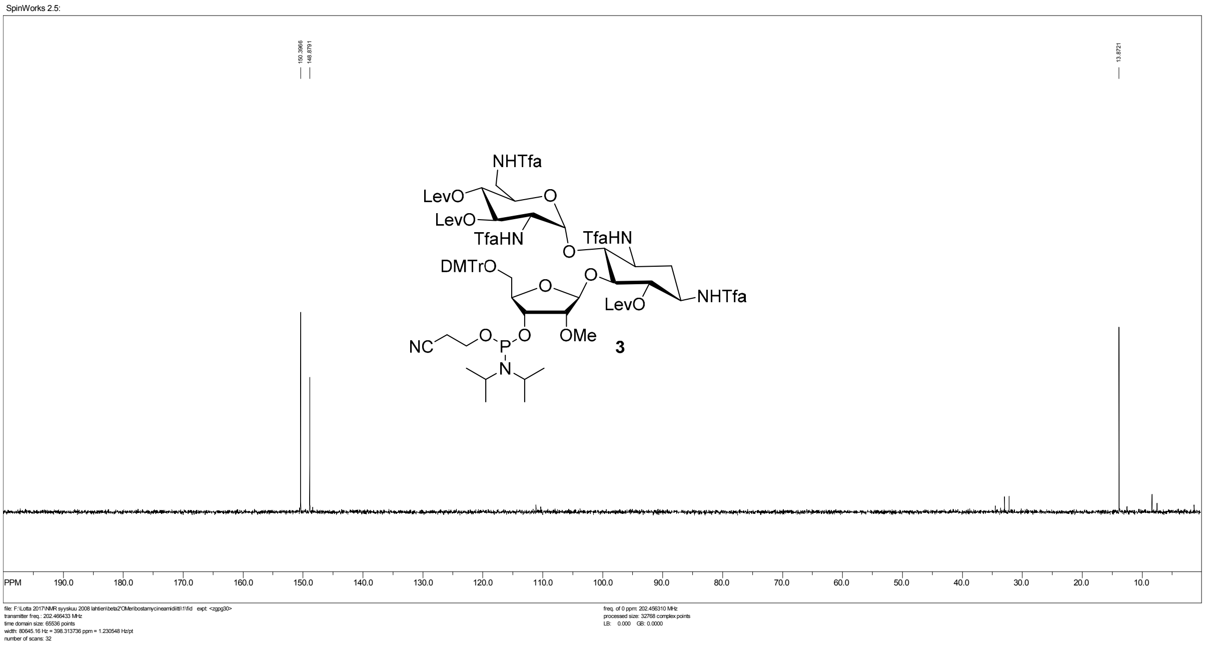

Figure S39. ^{31}P NMR (200 MHz, CD_3CN) spectrum of **3**.

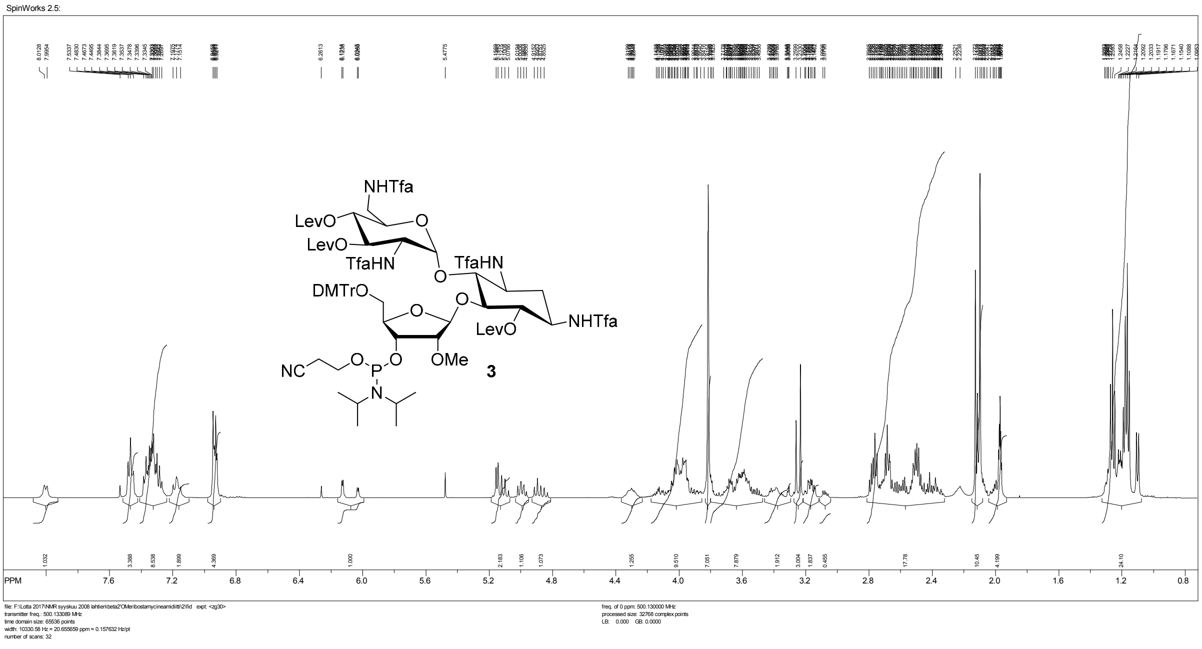

Figure S40. ^{1}H NMR (500 MHz, CD_3CN) spectrum of **3**.

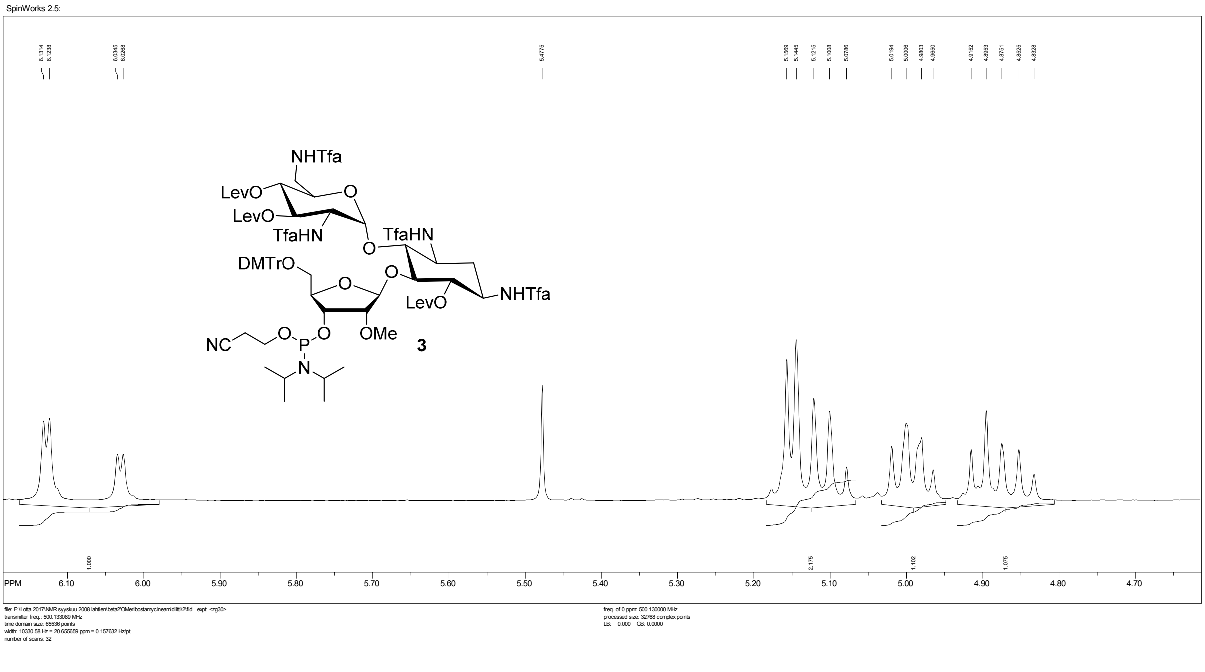

Figure S41. ^{1}H NMR (500 MHz, CD_3CN) spectrum of **3**.

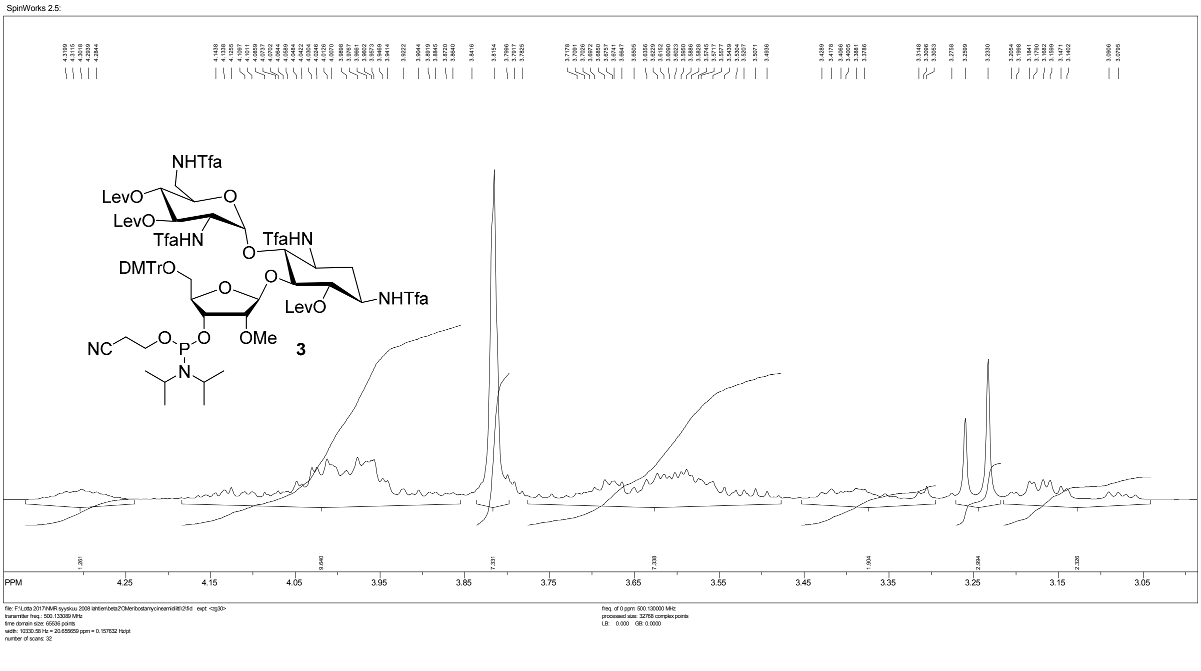

Figure S42. ^{1}H NMR (500 MHz, CD_3CN) spectrum of **3**.

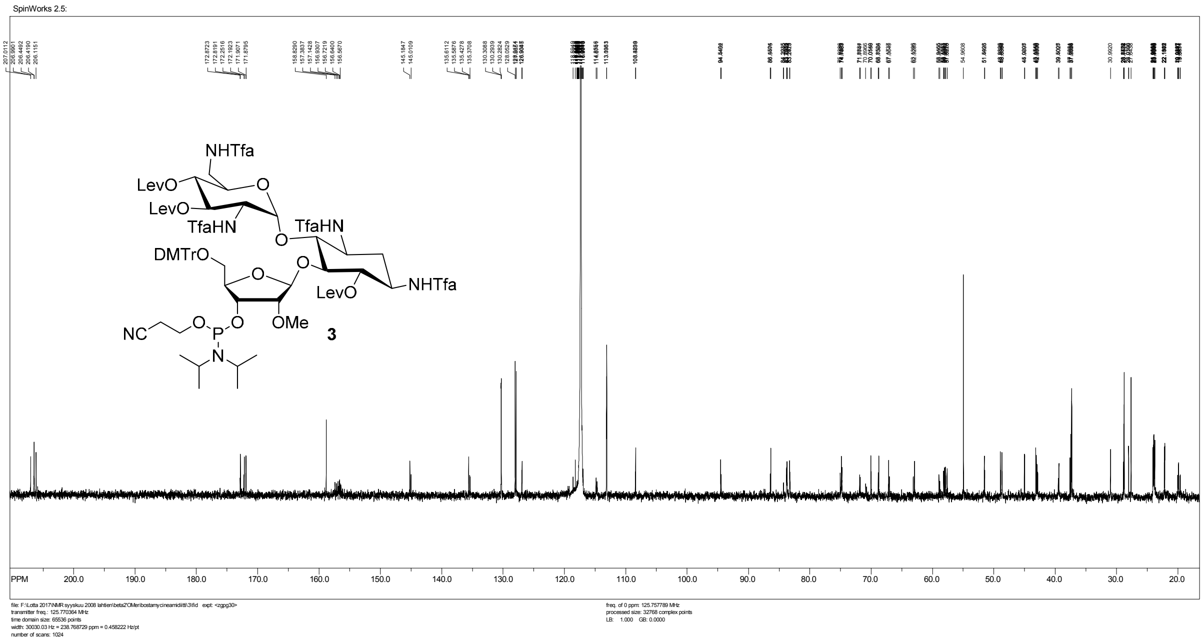

Figure S43. ^{13}C NMR (125 MHz, CD_3CN) spectrum of **3**.

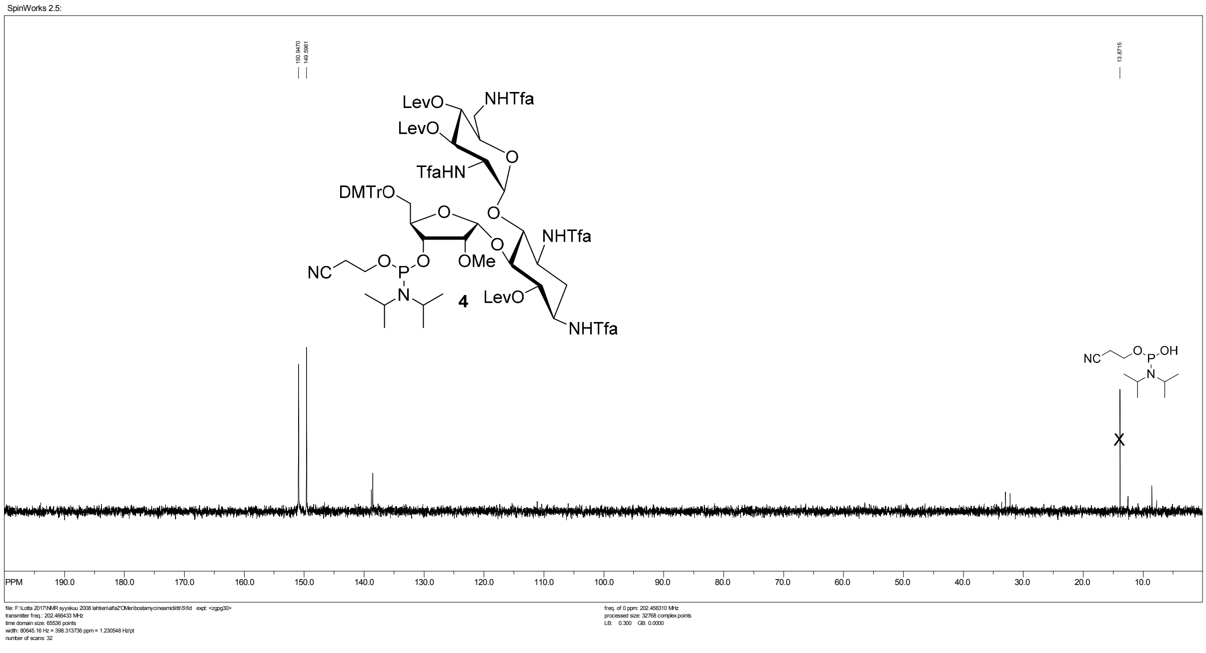

Figure S44. ^{31}P NMR (200 MHz, CD_3CN) spectrum of **4**.

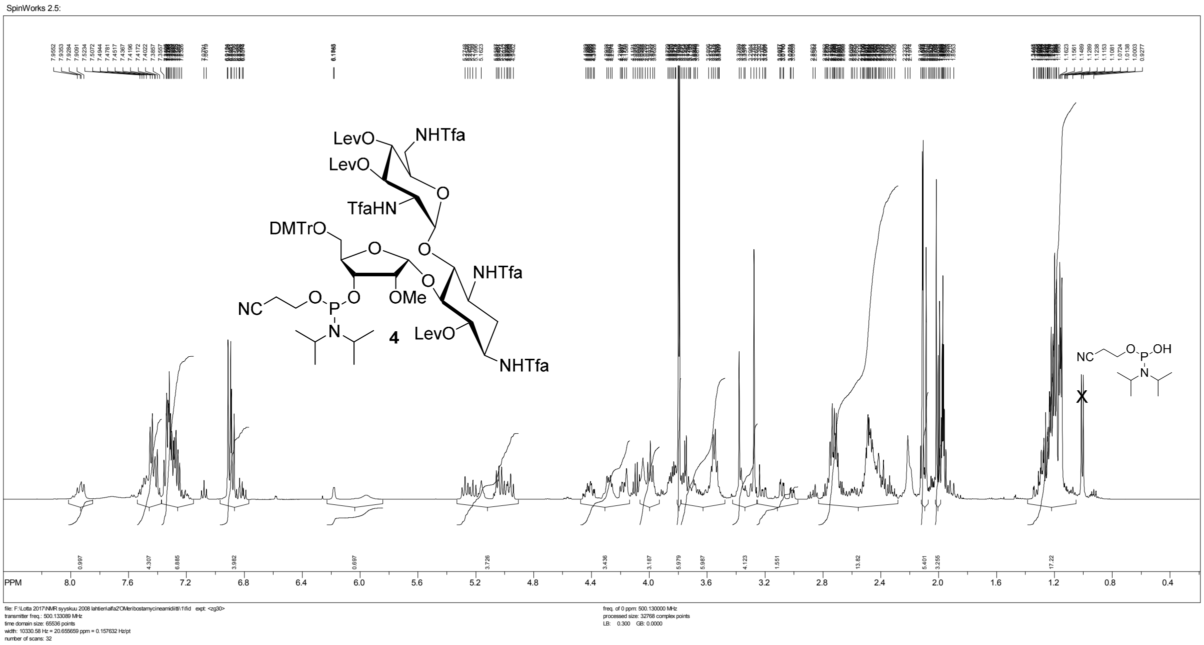

Figure S45. ^{1}H NMR (500 MHz, CD_3CN) spectrum of **4**.

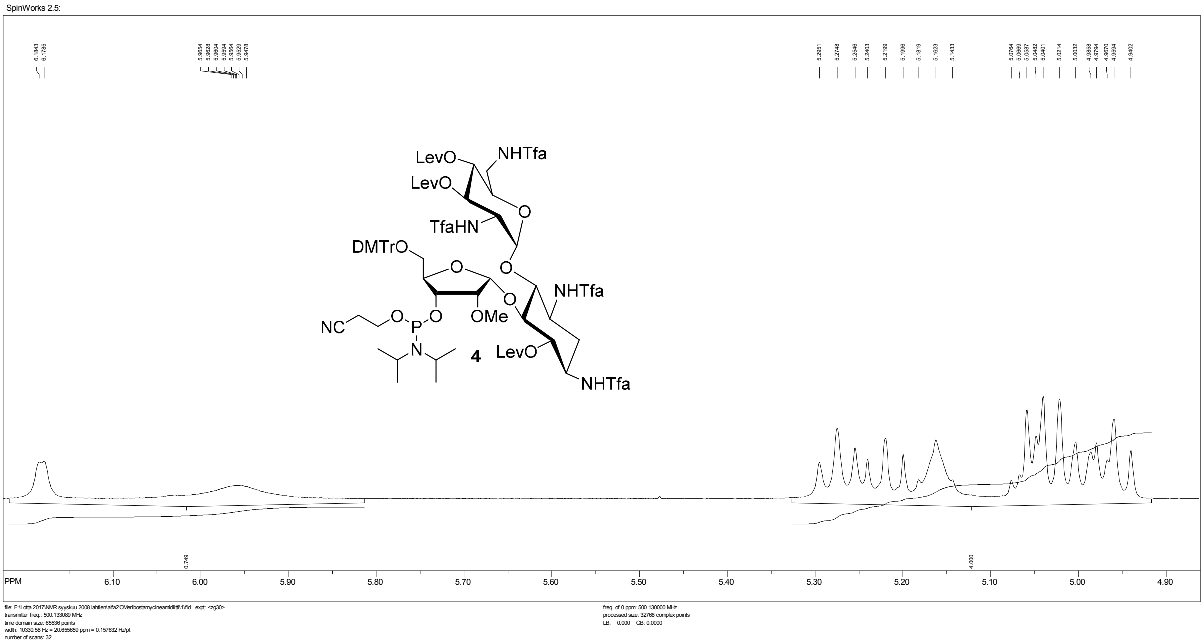

Figure S46. ^{1}H NMR (500 MHz, CD_3CN) spectrum of **4**.

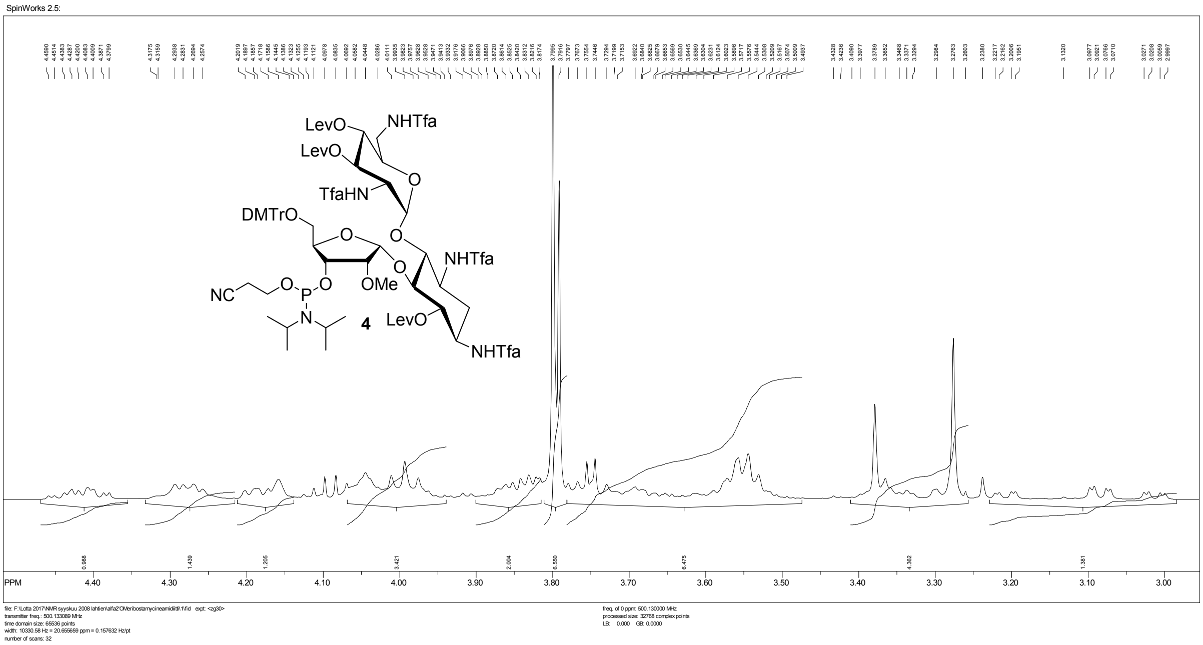

Figure S47. ^{1}H NMR (500 MHz, CD_3CN) spectrum of **4**.

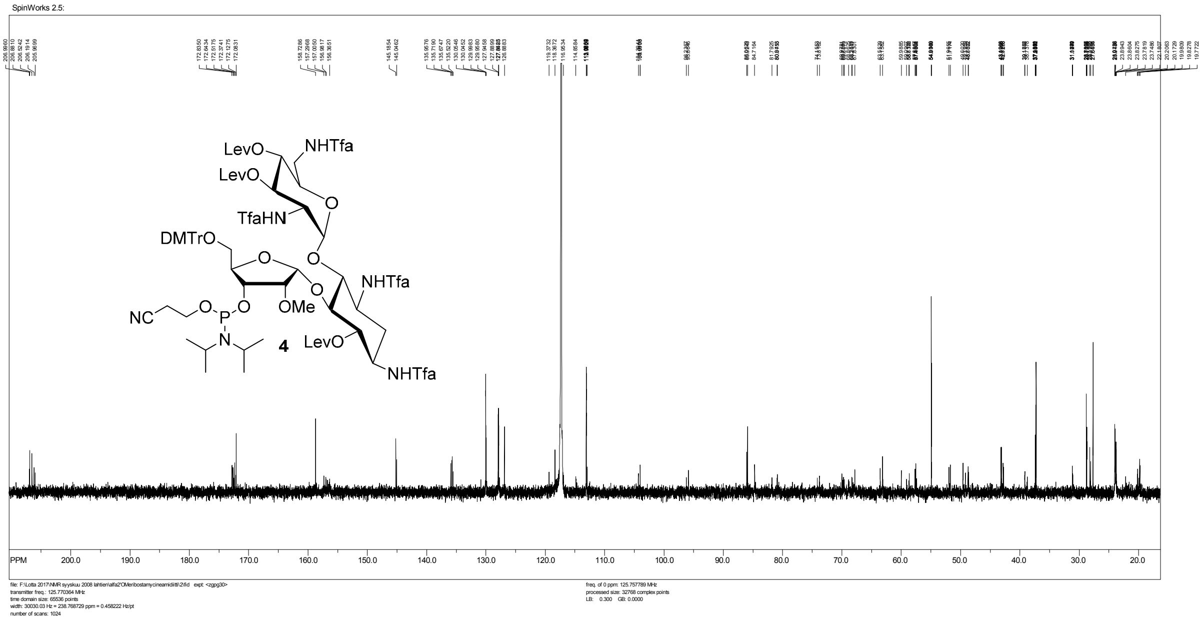

Figure S48. ^{13}C NMR (125 MHz, CD_3CN) spectrum of **4**.

Table S1. MS(ESI-TOF) data of the oligonucleotides.

	**ON1**	**ON2**	**ON4**	**ON5**	**ON6**	**ON7**	**ON8**
Observed molecular mass	3447.7	3447.7	10104.3	10635.3	10635.1	9653.6	9652.8
Calculated molecular mass	3448.4	3448.4	10103.9	10635.0	10635.0	9653.8	9653.8

[1] Notes: The observed molecular masses are calculated from $[(M\text{-}2H)/2]^{-2}$,(**ON1** and **ON2**) and $[(M\text{-}4H)/4]^{-4}$ (**ON3**-**ON8**)
